# Common Genetic Variation and Age of Onset of Anorexia Nervosa

**DOI:** 10.1016/j.bpsgos.2021.09.001

**Published:** 2021-09-20

**Authors:** Hunna J. Watson, Laura M. Thornton, Zeynep Yilmaz, Jessica H. Baker, Jonathan R.I. Coleman, Roger A.H. Adan, Lars Alfredsson, Ole A. Andreassen, Helga Ask, Wade H. Berrettini, Michael Boehnke, Ilka Boehm, Claudette Boni, Katharina Buehren, Josef Bulant, Roland Burghardt, Xiao Chang, Sven Cichon, Roger D. Cone, Philippe Courtet, Scott Crow, James J. Crowley, Unna N. Danner, Martina de Zwaan, George Dedoussis, Janiece E. DeSocio, Danielle M. Dick, Dimitris Dikeos, Christian Dina, Srdjan Djurovic, Monika Dmitrzak-Weglarz, Elisa Docampo-Martinez, Philibert Duriez, Karin Egberts, Stefan Ehrlich, Johan G. Eriksson, Geòrgia Escaramís, Tõnu Esko, Xavier Estivill, Anne Farmer, Fernando Fernández-Aranda, Manfred M. Fichter, Manuel Föcker, Lenka Foretova, Andreas J. Forstner, Oleksandr Frei, Steven Gallinger, Ina Giegling, Johanna Giuranna, Fragiskos Gonidakis, Philip Gorwood, Mònica Gratacòs, Sébastien Guillaume, Yiran Guo, Hakon Hakonarson, Joanna Hauser, Alexandra Havdahl, Johannes Hebebrand, Sietske G. Helder, Stefan Herms, Beate Herpertz-Dahlmann, Wolfgang Herzog, Anke Hinney, Christopher Hübel, James I. Hudson, Hartmut Imgart, Stephanie Jamain, Vladimir Janout, Susana Jiménez-Murcia, Ian R. Jones, Antonio Julià, Gursharan Kalsi, Deborah Kaminská, Jaakko Kaprio, Leila Karhunen, Martien J.H. Kas, Pamela K. Keel, James L. Kennedy, Anna Keski-Rahkonen, Kirsty Kiezebrink, Lars Klareskog, Kelly L. Klump, Gun Peggy S. Knudsen, Maria C. La Via, Stephanie Le Hellard, Marion Leboyer, Dong Li, Lisa Lilenfeld, Bochao Lin, Jolanta Lissowska, Jurjen Luykx, Pierre Magistretti, Mario Maj, Sara Marsal, Christian R. Marshall, Morten Mattingsdal, Ingrid Meulenbelt, Nadia Micali, Karen S. Mitchell, Alessio Maria Monteleone, Palmiero Monteleone, Richard Myers, Marie Navratilova, Ionna Ntalla, Julie K. O’Toole, Roel A. Ophoff, Leonid Padyukov, Jacques Pantel, Hana Papežová, Dalila Pinto, Anu Raevuori, Nicolas Ramoz, Ted Reichborn-Kjennerud, Valdo Ricca, Samuli Ripatti, Stephan Ripke, Franziska Ritschel, Marion Roberts, Alessandro Rotondo, Dan Rujescu, Filip Rybakowski, André Scherag, Stephen W. Scherer, Ulrike Schmidt, Laura J. Scott, Jochen Seitz, Yasmina Silén, Lenka Šlachtová, P. Eline Slagboom, Margarita C.T. Slof-Op ‘t Landt, Agnieszka Slopien, Sandro Sorbi, Beata Świątkowska, Alfonso Tortorella, Federica Tozzi, Janet Treasure, Artemis Tsitsika, Marta Tyszkiewicz-Nwafor, Konstantinos Tziouvas, Annemarie A. van Elburg, Eric F. van Furth, Esther Walton, Elisabeth Widen, Stephanie Zerwas, Stephan Zipfel, Andrew W. Bergen, Joseph M. Boden, Harry Brandt, Steven Crawford, Katherine A. Halmi, L. John Horwood, Craig Johnson, Allan S. Kaplan, Walter H. Kaye, James E. Mitchell, Catherine M. Olsen, John F. Pearson, Nancy L. Pedersen, Michael Strober, Thomas Werge, David C. Whiteman, D. Blake Woodside, Scott Gordon, Sarah Maguire, Janne T. Larsen, Richard Parker, Liselotte V. Petersen, Jennifer Jordan, Martin Kennedy, Tracey D. Wade, Andreas Birgegård, Paul Lichtenstein, Mikael Landén, Nicholas G. Martin, Preben Bo Mortensen, Gerome Breen, Cynthia M. Bulik

**Affiliations:** a Department of Psychiatry, University of North Carolina at Chapel Hill, Chapel Hill, North Carolina; b Department of Genetics, University of North Carolina at Chapel Hill, Chapel Hill, North Carolina; c Department of Nutrition, University of North Carolina at Chapel Hill, Chapel Hill, North Carolina; d Department of Psychiatry, Center for Neurobiology and Behavior, Philadelphia, Pennsylvania; e Department of Pediatrics, University of Pennsylvania Perelman School of Medicine, Philadelphia, Pennsylvania; f Center for Applied Genomics, Children’s Hospital of Philadelphia, Philadelphia, Pennsylvania; g Department of Biostatistics, University of Michigan, Ann Arbor, Michigan; h Life Sciences Institute and Department of Molecular and Integrative Physiology, University of Michigan, Ann Arbor, Michigan; i Department of Psychology, Michigan State University, Lansing, Michigan; j Department of Psychiatry, University of Minnesota, Minneapolis, Minnesota; k College of Nursing, Seattle University, Seattle, Washington; l Department of Clinical Psychology, the Chicago School of Professional Psychology, Washington, DC; m Program in Medical and Population Genetics, Broad Institute of MIT and Harvard, Cambridge, Massachusetts; n Stanley Center for Psychiatric Research, Broad Institute of MIT and Harvard, Cambridge, Massachusetts; o Biological Psychiatry Laboratory, McLean Hospital/Harvard Medical School, Boston, Massachusetts; p Women’s Health Sciences Division, National Center for PTSD, Boston, Massachusetts; q Department of Psychiatry, Boston University, Boston, Massachusetts; r Analytic and Translational Genetics Unit, Massachusetts General Hospital, Boston, Massachusetts; s The Center for Eating Disorders at Sheppard Pratt, Baltimore, Maryland; t Department of Psychology, Florida State University, Tallahassee, Florida; u HudsonAlpha Institute for Biotechnology, Huntsville, Alabama; v Kartini Clinic, Portland, Oregon; w Oregon Research Institute, Eugene, Oregon; x Center for Neurobehavioral Genetics, University of California at Los Angeles, Los Angeles, California; y Semel Institute for Neuroscience and Human Behavior, University of California at Los Angeles, Los Angeles, California; z David Geffen School of Medicine, University of California at Los Angeles, Los Angeles, California; aa Biorealm Research, Walnut, California; ab Department of Psychiatry, University of California San Diego, San Diego, California; ac Division of Psychiatric Genomics, Department of Psychiatry, and Genetics and Genomics Sciences, Icahn School of Medicine at Mount Sinai, New York; ad New York Presbyterian Hospital-Westchester Division, Weill Cornell Medical College of Cornell University, White Plains, New York; ae Eating Recovery Center, Denver, Colorado; af Department of Psychiatry and Behavioral Science, School of Medicine and Health Sciences, University of North Dakota, Fargo, North Dakota; ag Department of Psychology, Commonwealth University, Richmond, Virginia; ah College Behavioral and Emotional Health Institute, Commonwealth University, Richmond, Virginia; ai Department of Human and Molecular Genetics, Commonwealth University, Richmond, Virginia; aj School of Psychology, Curtin University, Perth, Western Australia, Australia; ak Division of Paediatrics, School of Medicine, The University of Western Australia, Perth, Western Australia, Australia; al Cancer Control Group, University of Queensland, Brisbane, Queensland, Australia; am QIMR Berghofer Medical Research Institute, University of Queensland, Brisbane, Queensland, Australia; an InsideOut Institute for Eating Disorders, The Charles Perkins Centre, the University of Sydney, Sydney, New South Wales, Australia; ao Sydney Local Health District, NSW Health, St. Leonards, New South Wales, Australia; ap College of Education, Psychology and Social Work, Flinders University, Adelaide, South Australia, Australia; aq National Centre for Register-based Research, Aarhus BSS, Aarhus, Denmark; ar Centre for Integrated Register-Based Research, Aarhus University, Aarhus, Denmark; as Lundbeck Foundation Initiative for Integrative Psychiatric Research, Aarhus, Denmark; at Department of Clinical Medicine, University of Copenhagen, Copenhagen, Denmark; au Department of Medical Epidemiology and Biostatistics, Karolinska Institutet, Stockholm, Sweden; av Institute of Environmental Medicine, Karolinska Institutet, Stockholm, Sweden; aw Department of Clinical Neuroscience, Karolinska Institutet, Stockholm, Sweden; ax Sahlgrenska Academy, University of Gothenburg, Gothenburg, Sweden; ay Institute of Neuroscience and Physiology, Gothenburg University, Gothenburg, Sweden; az Division of Rheumatology, Department of Medicine, Karolinska Institutet, Karolinska Institutet and Karolinska University Hospital, Solna, Sweden; ba Department of Psychological Medicine, Institute of Psychiatry, Psychology and Neuroscience, Social, Genetic and Developmental Psychiatry Centre, King’s College London, London, United Kingdom; bb Institute of Psychiatry, Psychology and Neuroscience, Social, Genetic and Developmental Psychiatry Centre, King’s College London, London, United Kingdom; bc National Institute for Health Research Biomedical Research Centre, King’s College London and South London and Maudsley National Health Service Foundation Trust, London, United Kingdom; bd National Centre for Mental Health, MRC Centre for Neuropsychiatric Genetics and Genomics, Cardiff University, Cardiff, United Kingdom; be Institute of Applied Health Sciences, School of Medicine, Medical Sciences and Nutrition, University of Aberdeen, Aberdeen, United Kingdom; bf Brain Center Rudolf Magnus, Department of Translational Neuroscience, University Medical Center Utrecht, the Netherlands; bg Department of Clinical Psychology, Faculty of Social Sciences, University Utrecht, Utrecht, the Netherlands; bh Altrecht Eating Disorders Rintveld, Zeist, the Netherlands; bi Groningen Institute for Evolutionary Life Sciences, University of Groningen, Groningen, the Netherlands; bj Department of Biomedical Data Science, Leiden University Medical Centre, Leiden, the Netherlands; bk Department of Psychiatry, Leiden University Medical Centre, Leiden, the Netherlands; bl Rivierduinen Eating Disorders Ursula, Leiden, the Netherlands; bm Department of Psychiatry, Erasmus MC, University Medical Center Rotterdam, Rotterdam, the Netherlands; bn Zorg op Orde, Delft, the Netherlands; bo NORMENT Centre, Division of Mental Health and Addiction, Oslo University Hospital, Oslo, Norway; bp NORMENT Centre, Department of Clinical Science, University of Oslo, Oslo, Norway; bq NORMENT Centre, Institute of Clinical Medicine, University of Oslo, Oslo, Norway; br Institute of Clinical Medicine, University of Oslo, Oslo, Norway; bs Nic Waals Institute, Lovisenberg Diaconal Hospitaland, Oslo, Norway; bt Department of Mental Disorders, Norwegian Institute of Public Health, Oslo, Norway; bu Department of Medical Genetics, University of Bergen, Bergen, Norway; bv Department of Clinical Science, K.G. Jebsen Centre for Psychosis Research, Norwegian Centre for Mental Disorders Research, University of Bergen, Bergen, Norway; bw Dr. Einar Martens Research Group for Biological Psychiatry, Center for Medical Genetics and Molecular Medicine, Haukeland University Hospital, Bergen, Norway; bx Department of Clinical Medicine, Laboratory Building, Haukeland University Hospital, Bergen, Norway; by Division of Psychological and Social Medicine and Developmental Neurosciences, Faculty of Medicine, Technische Universität Dresden, Dresden, Germany; bz Eating Disorders Research and Treatment Center, Department of Child and Adolescent Psychiatry, Faculty of Medicine, Technische Universität Dresden, Dresden, Germany; ca Department of Child and Adolescent Psychiatry, Psychosomatics and Psychotherapy, RWTH Aachen University, Aachen, Germany; cb Department of Psychosomatic Medicine and Psychotherapy, Hannover Medical School, Hannover, Germany; cc Department of Child and Adolescent Psychiatry, Klinikum Frankfurt/Oder, Frankfurt, Germany; cd Institute of Neuroscience and Medicine, Research Center Jülich, Jülich, Germany; ce Department of Child and Adolescent Psychiatry, Psychosomatics and Psychotherapy, University Hospital of Würzburg, Centre for Mental Health, Würzburg, Germany; cf Department of Psychiatry and Psychotherapy, Ludwig-Maximilians-University, Munich, Germany; cg Schön Klinik Roseneck Affiliated With the Medical Faculty of the University of Munich, Prien, Germany; ch Department of Child and Adolescent Psychiatry, University Hospital Münster, Münster, Germany; ci Institute of Human Genetics, University of Bonn, School of Medicine & University Hospital Bonn, Bonn, Germany; cj Centre for Human Genetics, University of Marburg, Marburg, Germany; ck Department of Psychiatry, Psychotherapy and Psychosomatics, Martin-Luther-University Halle-Wittenberg, Halle, Germany; cl Department of Child and Adolescent Psychiatry, Psychosomatics and Psychotherapy, University Hospital Essen, University of Duisburg-Essen, Essen, Germany; cm Department of General Internal Medicine and Psychosomatics, Heidelberg University Hospital, Heidelberg University, Heidelberg, Germany; cn Eating Disorders Unit, Parklandklinik, Bad Wildungen, Germany; co Department of Psychiatry and Psychotherapy, Charité – Universitätsmedizin, Berlin, Germany; cp Institute of Medical Statistics, Computer and Data Sciences, Jena University Hospital – Friedrich Schiller University Jena, Jena, Germany; cq Department of Internal Medicine VI, Psychosomatic Medicine and Psychotherapy, University Medical Hospital Tuebingen, Tuebingen, Germany; cr Centre of Excellence for Eating Disorders, University Tuebingen, Tuebingen, Germany; cs INSERM 1266, Institute of Psychiatry and Neuroscience of Paris, Paris, France; ct INSERM 1124, Université de Paris, Paris, France; cu GHU Paris Psychiatrie et Neurosciences, CMME, Paris, France; cv CMME (GHU Paris Psychiatrie et Neurosciences), Hôpital Sainte Anne, Paris, France; cw Department of Emergency Psychiatry and Post-Acute Care, CHRU Montpellier, University of Montpellier, Montpellier, France; cx L’institut du thorax, INSERM, CNRS, UNIV Nantes, Nantes, France; cy Inserm U955, Institut Mondor de recherches Biomédicales, Laboratoire, Neuro-Psychiatrie Translationnelle, and Fédération Hospitalo-Universitaire de Précision Médecine en Addictologie et Psychiatrie, University Paris-Est-Créteil, Créteil, France; cz Department of Pediatrics and Adolescent Medicine, First Faculty of Medicine, Charles University and General University Hospital, Prague, Czech Republic; da Department of Psychiatry, First Faculty of Medicine, Charles University and General University Hospital, Prague, Czech Republic; db Eating Disorders Unit, Department of Psychiatry, First Faculty of Medicine, Charles University and General University Hospital, Prague, Czech Republic; dc Department of Biochemistry and Molecular Biology, Institute of Organic Chemistry and Biochemistry, Prague, Czech Republic; dd Department of Cancer, Epidemiology and Genetics, Masaryk Memorial Cancer Institute, Brno, Czech Republic; de Faculty of Health Sciences, Palacky University, Olomouc, Czech Republic; df Institute of Medical Genetics and Pathology, University Hospital Basel, Basel, Switzerland; dg Department of Biomedicine, University of Basel, Basel, Switzerland; dh Department of Psychiatry, University of Lausanne-University Hospital of Lausanne, Lausanne, Switzerland; di Department of Psychiatry, Faculty of Medicine, University of Geneva, Geneva, Switzerland; dj Department of Pediatrics Gynaecology and Obstetrics, University of Geneva, Geneva, Switzerland; dk Department of Nutrition and Dietetics, Harokopio University, Athens, Greece; dl Adolescent Health Unit, Second Department of Pediatrics, Athens, Greece; dm Pediatric Intensive Care Unit, “P. & A. Kyriakou” Children's Hospital, University of Athens, Athens, Greece; dn First Department of Psychiatry, National and Kapodistrian University of Athens, Medical School, Eginition Hospital, Athens, Greece; do Department of Psychiatric Genetics, Poznan University of Medical Sciences, Poznan, Poland; dp Department of Adult Psychiatry, Poznan University of Medical Sciences, Poznan, Poland; dq Department of Psychiatry, Poznan University of Medical Sciences, Poznan, Poland; dr Department of Child and Adolescent Psychiatry, Poznan University of Medical Sciences, Poznan, Poland; ds Department of Cancer Epidemiology and Prevention, M. Sklodowska-Curie National Research Institute of Oncology, Warsaw, Poland; dt Department of Environmental Epidemiology, the Reference Center for Asbestos Exposure and Health Risk Assessment, Lódź, Poland; du Barcelona Institute of Science and Technology, Barcelona, Spain; dv Universitat Pompeu Fabra, Barcelona, Spain; dw Centro de Investigación Biomédica en Red en Epidemiología y Salud Pública, Barcelona, Spain; dx Genomics and Disease, Bioinformatics and Genomics Programme, Centre for Genomic Regulation, Barcelona, Spain; dy Department of Psychiatry, University Hospital Bellvitge-IDIBELL and CIBEROBN, Barcelona, Spain; dz Department of Clinical Sciences, School of Medicine, University of Barcelona, Barcelona, Spain; ea Rheumatology Research Group, Vall d’Hebron Research Institute, Barcelona, Spain; eb Department of Public Health Solutions, National Institute for Health and Welfare, Helsinki, Finland; ec Folkhälsan Research Center, Helsinki, Finland; ed Department of General Practice and Primary Health Care, University of Helsinki and Helsinki University Hospital, Helsinki, Finland; ee Department of Adolescent Psychiatry, Helsinki University Hospital and University of Helsinki, Helsinki, Finland; ef Institute for Molecular Medicine Finland, Helsinki Institute of Life Science, University of Helsinki, Helsinki, Finland; eg Clinicum, Department of Public Health, University of Helsinki, Helsinki, Finland; eh Department of Adolescent Psychiatry, University of Helsinki, Helsinki, Finland; ei Department of Public Health, University of Helsinki, Helsinki, Finland; ej Department of Biometry, University of Helsinki, Helsinki, Finland; ek Institute for Molecular Medicine Finland, Helsinki Institute of Life Science, Helsinki, Finland; el Institute of Public Health and Clinical Nutrition, Department of Clinical Nutrition, University of Eastern Finland, Kuopio, Finland; em Estonian Genome Center, Institute of Genomics, University of Tartu, Tartu, Estonia; en Department of Surgery, University of Toronto, Toronto, Ontario, Canada; eo Institute of Medical Science, University of Toronto, Toronto, Ontario, Canada; ep Department of Psychiatry, University of Toronto, Toronto, Ontario, Canada; eq McLaughlin Centre, University of Toronto, Toronto, Ontario, Canada; er Centre for Addiction and Mental Health, Toronto, Ontario, Canada; es Centre for Mental Health, University Health Network, Toronto, Ontario, Canada; et Program for Eating Disorders, University Health Network, Toronto, Ontario, Canada; eu Department of Paediatric Laboratory Medicine, the Hospital for Sick Children, Toronto, Ontario, Canada; ev Department of Genetics and Genome Biology and the Center for Applied Genomics, the Hospital for Sick Children, Toronto, Ontario, Canada; ew BESE Division, King Abdullah University of Science and Technology, Thuwal, Saudi Arabia; ex Department of Psychiatry, University of Campania “Luigi Vanvitelli,” Naples, Italy; ey Department of Medicine, Surgery and Dentistry “Scuola Medica Salernitana”, University of Salerno, Salerno, Italy; ez Department of Neuroscience, Psychology, Drug Research and Child Health, University of Florence, Florence, Italy; fa Department of Health Science, University of Florence, Florence, Italy; fb IRCSS Fondazione Don Carlo Gnocchi, Florence, Italy; fc Department of Psychiatry, Neurobiology, Pharmacology, and Biotechnologies, University of Pisa, Pisa, Italy; fd Department of Psychiatry, University of Perugia, Perugia, Italy; fe Department of Psychological Medicine, University of Otago, Christchurch, New Zealand; ff Biostatistics and Computational Biology Unit, University of Otago, Christchurch, New Zealand; fg Pathology and Biomedical Science, University of Otago, Christchurch, New Zealand; fh Clinical Research Unit, Canterbury District Health Board, Christchurch, New Zealand; fi Brain Sciences Department, Stremble Ventures, Limassol, Cyprus

**Keywords:** Age of onset, Anorexia nervosa, Early-onset, Genetic risk score, Genetics, GWAS, Menarche, Mendelian randomization, Puberty

## Abstract

**Background:**

Genetics and biology may influence the age of onset of anorexia nervosa (AN). The aims of this study were to determine whether common genetic variation contributes to age of onset of AN and to investigate the genetic associations between age of onset of AN and age at menarche.

**Methods:**

A secondary analysis of the Psychiatric Genomics Consortium genome-wide association study (GWAS) of AN was performed, which included 9335 cases and 31,981 screened controls, all from European ancestries. We conducted GWASs of age of onset, early-onset AN (<13 years), and typical-onset AN, and genetic correlation, genetic risk score, and Mendelian randomization analyses.

**Results:**

Two loci were genome-wide significant in the typical-onset AN GWAS. Heritability estimates (single nucleotide polymorphism–*h*^2^) were 0.01–0.04 for age of onset, 0.16–0.25 for early-onset AN, and 0.17–0.25 for typical-onset AN. Early- and typical-onset AN showed distinct genetic correlation patterns with putative risk factors for AN. Specifically, early-onset AN was significantly genetically correlated with younger age at menarche, and typical-onset AN was significantly negatively genetically correlated with anthropometric traits. Genetic risk scores for age of onset and early-onset AN estimated from independent GWASs significantly predicted age of onset. Mendelian randomization analysis suggested a causal link between younger age at menarche and early-onset AN.

**Conclusions:**

Our results provide evidence consistent with a common variant genetic basis for age of onset and implicate biological pathways regulating menarche and reproduction.

Anorexia nervosa (AN) is an eating disorder characterized by starvation, low body mass index (BMI), and a morbid fear of weight gain, affecting 0.9% to 1.4% of females and 0.1% to 0.3% of males (1,2). The etiology involves a complex interplay between genetics and the environment (3). Twin-based studies report a heritability of 50% to 60% (4). Common genetic polymorphisms account for a substantial portion of this heritability (single nucleotide polymorphism [SNP]-*h*^2^ = 11%–17%) (5). Peak age of onset is between 16 and 22 years in community-based epidemiological research (1) and 14 to 19 years in clinical populations (6), and onset after age 25 is atypical (7). To our knowledge, there are no genome-wide association study (GWAS) or heritability studies of age of onset of AN, and the factors that contribute to earlier rather than later onset are unknown (7).

The Psychiatric Genomics Consortium (PGC) GWAS of AN identified eight genomic regions associated with the risk of lifetime AN and implicated a psychiatric and metabolic etiology (5). Candidate gene studies have suggested that polymorphisms in serotonergic and appetite-regulating genes might be associated with age of onset (8, 9, 10, 11). However, candidate gene studies generally have been subject to important criticisms, including nonreplication. In illnesses such as schizophrenia and bipolar disorder, a higher genetic burden predicts earlier onset and age of onset for some psychiatric traits aggregates in families (12, 13, 14, 15). The Brainstorm Consortium combined molecular genetic data from 10 psychiatric disorders including AN and found a modest, significant correlation linking earlier age of onset to higher heritability (16). Meanwhile, twin studies of eating disorder symptoms suggest that genetic contributions change across development such that genetic effects explain negligible variance prepuberty and increase substantially in peripuberty (17). Because genetic factors influence AN risk, the first aim of this study is to investigate whether common genetic polymorphisms account for variation in age of onset (aim 1).

Although GWAS efforts have made tremendous contributions to our understanding of the genetic etiology of AN, phenotypic and genetic heterogeneity can hinder discovery of the genetic architecture of psychiatric traits (18). Insight can be improved by leveraging investigations of etiologically homogeneous illness subphenotypes, such as early-onset presentations. Differences in clinical presentation of AN by age are evident, although not well established because of limited research, with early-onset cases displaying predominantly non–binge/purge profiles, a faster rate of weight loss, less endorsement of psychological symptoms, more favorable long-term outcomes, and a higher male prevalence than typical-onset presentations (19, 20, 21, 22). The second aim of this study is to examine a subphenotype of AN, specifically early-onset, to aid in discovering the genetics and biology of AN and age of onset (aim 2).

Early pubertal timing has long been cited as a risk factor for AN, particularly early-onset AN, but the evidence base is weak and observational, and methodological issues complicate investigation (17). The relatively low prevalence of AN impedes prospective designs, and nutritional deficiencies in AN arrest pubertal development and complicate estimates of the causal influence of pubertal traits. Genetic designs, such as Mendelian randomization, can interrogate causality under specific assumptions while avoiding these measurement confounds. Population-based twin research has shown that shared genetic factors influence liability to earlier menarche and disordered eating (23). However, a significant genetic correlation between age at menarche (i.e., a commonly used measure of puberty timing) and AN was not evident in the largest AN GWAS to date (5). Large-scale genomic and phenotypic data collections and new analytic methods (i.e., genetic risk score [GRS] analysis, Mendelian randomization) have become available and present an excellent opportunity to examine whether puberty timing may be a causal risk factor for AN risk and age of onset (aim 3).

## Methods and Materials

### Design and Participants

This is a secondary analysis of individual-level data from a GWAS of AN (5), which we refer to as the parent study. Cohorts from the parent study were included here if they had cases with age of onset data. This resulted in 13 cohorts and 55% of cases (*N* = 9335) and 58% of controls (*N* = 31,981) included in this study from the parent study of 33 cohorts, 16,992 cases, and 55,525 controls (Table S1 in Supplement 2). For some secondary analyses using AN risk as a phenotype, all cases from the 13 cohorts above were included, resulting in 11,632 cases and 31,981 controls for those analyses. More details on recruitment, phenotyping, DNA collection, and genotyping are provided in Supplement 1 and other publications (5,24,25). Supplemental Methods in Supplement 1 and Table S2 in Supplement 2 provide phenotyping information for age of onset and early-onset AN, which was characterized as onset before age 13 years.

### GWAS of Age of Onset of AN, Early-Onset AN, and Typical-Onset AN

Three GWASs were conducted: 1) a within-case GWAS on age of onset; 2) a case-control GWAS that stratified a subset of cases by the subphenotype of early-onset AN and compared these with ancestrally matched controls; and 3) a case-control GWAS of typical-onset AN for comparative purposes with respect to the genetic correlations and other secondary analyses, as detailed later (Table S3 in Supplement 2).

Quality control of genotype data is described in Supplement 1. GWASs were conducted using RICOPILI (26). Samples were of European ancestry, and genotypes were imputed to the 1000 Genomes reference (27). The first five principal components were included to capture ancestry-based population stratification. Linear regression for age of onset and logistic regression for early-onset AN and typical-onset AN were carried out on imputed variant dosages using additive models to test for associations between the markers and the phenotypes. Cohort-level GWAS analyses were combined with fixed-effects meta-analysis (including variants with imputation INFO scores > 0.7). The standard genome-wide cutoff (*p* < 5 × 10^−8^) was anticonservative, given three GWASs; therefore, results were interpreted at a Bonferroni-corrected threshold (*p* < 1.67 × 10^−8^). The GWAS of age of onset had >80% statistical power to detect genetic effects with 0.45% of the variance explained (*R*^2^), or βs between 1.06 and 1.59 (at minor allele frequency 0.05–0.5); the GWAS of early-onset AN had >80% power to detect an odds ratio (OR) between 1.32 and 1.70 (at minor allele frequency 0.05–0.5, assuming a lifetime prevalence of 0.1%) (28); and the GWAS of typical-onset AN had >80% power to detect an OR between 1.14 and 1.31 (at minor allele frequency 0.05–0.5, assuming a lifetime prevalence of 0.9%–4%) (29). Common variant heritability was estimated with linkage disequilibrium score regression (LDSC) (30) and the genomic-relatedness-based restricted maximum-likelihood (GREML) approach (31) implemented in GCTA (31). GREML analyses had 80% power to detect SNP-*h*^2^s ≥ 0.1 for age of onset, 0.07 for early-onset AN (liability scale), and 0.03 for typical-onset AN (liability scale) (32). Genetic correlation analyses were conducted on LD Hub (33), and genetic correlation analyses with two AN GWASs (5,34) not on LD Hub and between early- and typical-onset AN used LDSC (30,33). Gene mapping and tissue expression analyses were performed with FUMA (35).

### GRSs as Predictors of Age of Onset of AN (GRS_age of onset,_ GRS_early-onset AN_, and GRS_AN_)

GRS analyses were conducted for aim 1 to investigate the evidence for a common variant–based genetic etiology. A GRS represents the combined effect of risk alleles carried by the individual and more powerfully predicts a complex trait than a single-SNP association analysis. Three sets of GRS at various *p* thresholds (*p*_T_s) were calculated for each individual with PRSice-2 (36) using the leave-one-cohort-out approach: 1) GRS_age of onset_ using the age of onset GWAS from this study, 2) GRS_AN_ using the GWAS results of the parent study (5), and 3) GRS_early-onset AN_ using the case-control GWAS for early-onset AN from this study (Supplemental Methods in Supplement 1). Linear regression quantified the association between GRS_age of onset_, GRS_early-onset AN_, and GRS_AN_ with age of onset with β and *R*^2^. Cohort-level analyses were combined with fixed-effects meta-analysis. *p* values were corrected using the false discovery rate (FDR) procedure (37).

### GRS_age at menarche_ as a Predictor of Age of Onset of AN, Early-Onset AN, Typical-Onset AN, and AN Risk

Large-scale genetic data have shown moderate to strong correlations between age at menarche and pubertal milestones across sexes, supporting the choice to capture puberty timing with age at menarche (38). GRS_age at menarche_ was calculated using the summary statistics from Day *et al.* (38). GRS analyses were carried out using the procedure above to address aim 3 (Supplemental Methods in Supplement 1).

### Causal Associations Between Puberty Timing and Age of Onset of AN, Early-Onset AN, Typical-Onset AN, and AN Risk

Mendelian randomization estimated the causal association between age at menarche and age of onset, early-onset AN, typical-onset AN, and AN risk, per aim 3. We used known genetic variants in GWAS summary statistics as instruments (5,38). Analyses were conducted with the TwoSampleMR package of MR-Base (39). We used the inverse-variance weighted estimator, which meta-analyzes the SNP-specific Wald estimates, and sensitivity approaches were applied (Supplement 1). Power calculations implied >80% power to detect a β of 1.01 for age at menarche on AN age of onset, OR of 0.90 for age at menarche on early-onset AN, OR of 1.16 for age at menarche on typical-onset AN, OR of 0.90 for age at menarche on AN risk, and β ≤ 0.99 for AN risk on age at menarche (40). Age at menarche was hypothesized to be inversely associated with early-onset AN and AN risk.

## Results

### Age of Onset Phenotype Summary

Table S1 in Supplement 2 describes the cohorts. The mean age of onset among the 9335 AN cases (99% female) in the 13 cohorts was 15.91 years (SD = 4.29, range 5–58), and approximately 15% had early-onset AN. A density plot of age of onset is shown in Figure S1 in Supplement 1.

### GWASs of Age of Onset, Early-Onset AN, and Typical-Onset AN

The GWAS of age of onset (13 cohorts, 9335 cases) yielded no SNPs with *p* values < 1.67 × 10^−8^ (Figure S2 in Supplement 1). The SNP with the lowest *p* value was rs146976977 (*p* = 1.85 × 10^−7^). The phenotypic variance explained by GRS_age of onset_ was *R*^2^ = 0.13%. SNP-*h*^2^ was 0.04 (SE = 0.05) with LDSC and 0.01 (SE = 0.02) with GREML.

No significant loci (*p* < 1.67 × 10^−8^) were observed for the GWAS of early-onset AN (5 cohorts, 1269 cases and 25,042 controls), although one false positive was observed at the standard genome-wide significance threshold (Figure S2 and Supplemental Results in Supplement 1). The false-positive designation was given because the SNP had no nearby linkage disequilibrium friends, an unrealistically large OR, was not genotyped (INFO score = 0.74), is low frequency, occurs in predominantly European populations, and based on the small sample size. GRS_early-onset AN_ explained 0.85% of the variance (liability scale *R*^2^), and observed scale SNP-*h*^2^ was 0.08 (SE = 0.02) with LDSC and 0.11 (SE = 0.01) with GREML. Liability scale SNP-*h*^2^ was 0.16–0.19 (SE = 0.04) with LDSC and 0.21–0.25 (SE = 0.02) with GREML, assuming a lifetime prevalence of 0.1% to 0.3% (28).

The GWAS of typical-onset AN (5 cohorts, 6998 cases and 25,042 controls) revealed two genome-wide significant loci (chromosome 3, rs3821875, OR = 1.21, 95% CI 1.14 to 1.29, *p* = 5.38 × 10^−10^; chromosome 9, rs4641158, OR = 1.23, 95% CI 1.15 to 1.31, *p* = 2.63 × 10^−9^) (Figure S2 in Supplement 1). The first was the top locus in the parent study and is complex and multigenic (i.e., >100 genes) with many chromatin and expression quantitative trait loci interactions. The second single-gene locus was not significant in the parent study and encodes *CNTLN* (centlein, centrosomal protein), which organizes microtubules (41) and is expressed in ovarian cells (42). Gene expression was most enriched in brain tissues, although no tissue expression *p* values (Figure S3 in Supplement 1), nor gene sets (Table S4 in Supplement 2), were Bonferroni significant. GRS_typical-onset AN_ explained 0.25% of the variance (liability scale *R*^2^). Observed scale SNP-*h*^2^ was 0.21 (SE = 0.02) with LDSC and 0.19 (SE = 0.01) with GREML, and liability scale SNP-*h*^2^ assuming a lifetime prevalence of 0.9% to 4% (1,43,44) was 0.17–0.25 (SE = 0.02) with LDSC and 0.15–0.22 (SE = 0.02) with GREML.

The allelic effects at the eight genome-wide significant loci in the AN risk GWAS were investigated in the early- and typical-onset GWASs, and allelic effects were similar (Tables S5a–c in Supplement 2).

### Genetic Correlations of Age of Onset, Early-Onset AN, and Typical-Onset AN

The genetic correlation between early- and typical-onset AN did not differ significantly from unity, *r*_g_ = 0.81 (SE = 0.12). Owing to the low heritability of age of onset, the quantitative trait, we had insufficient power to explore genetic correlations with other traits, and none reached even nominal significance (*p* < .05).

We investigated genetic correlations between early-onset AN and 62 traits prioritized from six categories based on previous evidence from AN (5,17): psychiatric (i.e., schizophrenia, major depressive disorder), anthropometric (i.e., weight, height), glycemic (i.e., type 2 diabetes, insulin resistance), lipid related (i.e., high-density lipoprotein cholesterol, triglycerides, leptin), reproductive (i.e., age at menarche, age at menopause), and education and intelligence (i.e., IQ, college completion). Nominally significant (*p* < .05) genetic correlations were observed with eight traits (Table S6 in Supplement 2). Ranked in order of increasing *p* values, these were reproductive, education, glycemic, and lipid-related traits, but none were significant after FDR correction (37). We also considered the genetic correlations between early-onset AN, all 700+ traits on LD Hub, and previous GWAS of AN (Table S7 in Supplement 2). FDR-significant correlations were observed between early-onset AN and the three previous AN GWASs and a UK Biobank question on whether help was sought from a psychiatrist for nerves, anxiety, tension, or depression. The genetic correlations for typical-onset AN are in Table S8 in Supplement 2.

**Figure 1:**
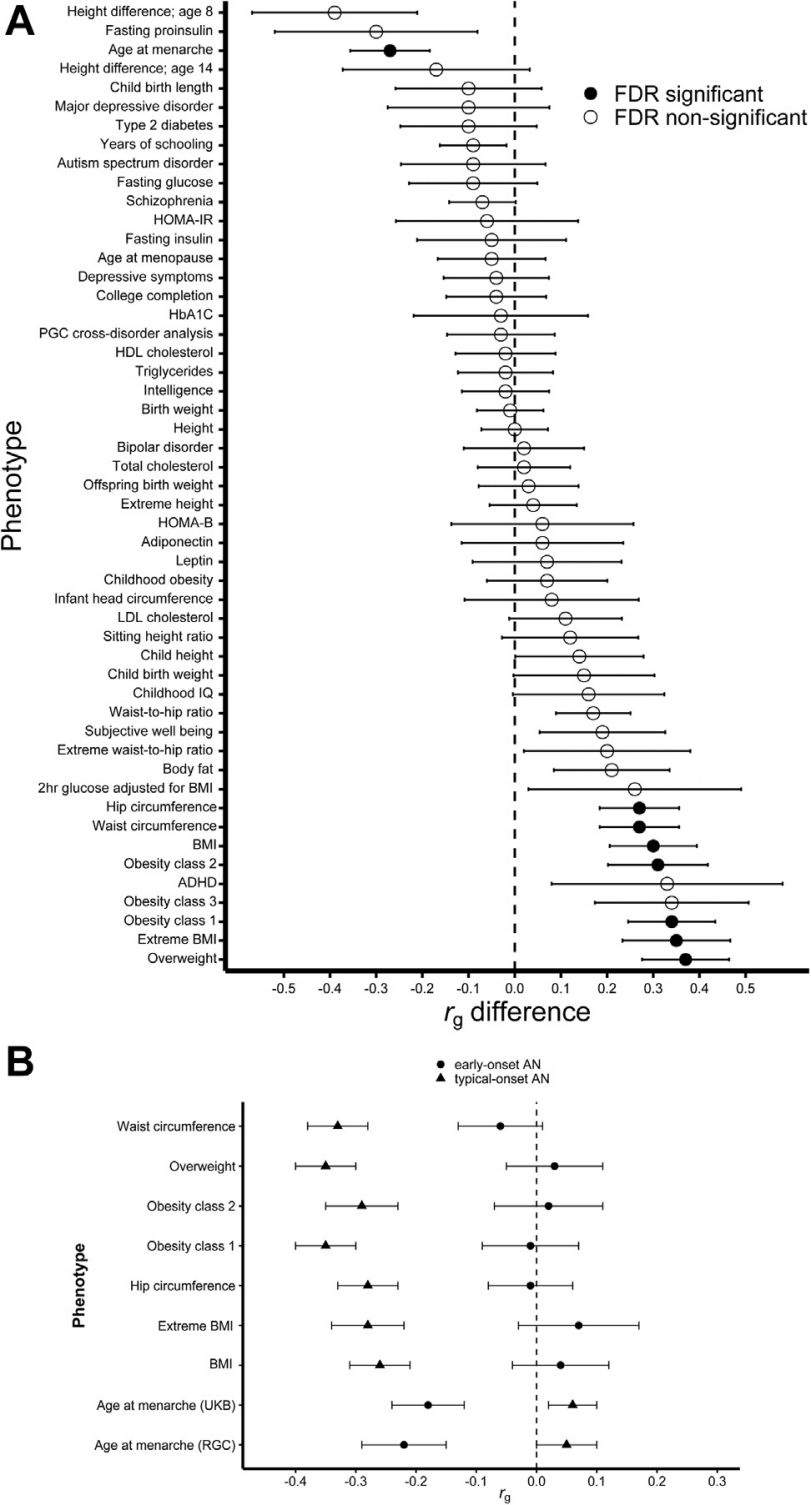
Early-onset and typical-onset AN show significantly different genetic correlation patterns with risk and comorbid traits. FDR-significant differences in genetic correlations were detected in two categories—anthropometric and reproductive—within six previously identified categories of risk or comorbid traits of interest for AN. **(A)** Sixty-two phenotypes were tested; duplicate phenotypes are not plotted (for duplicate phenotypes, we prioritized published summary statistics or the *r*_g_ difference with the lowest SE). Full results are shown in Table S9 in Supplement 2. **(B)** Shows the *r*_g_s between the phenotypes and the age of onset subphenotypes. The error bars in both plots represent the SE. ADHD, attention-deficit/hyperactivity disorder; AN, anorexia nervosa; BMI, body mass index; FDR, false discovery rate; HbA1C, hemoglobin A_1c_; HDL, high-density lipoprotein; HOMA-B, homeostatic model assessment for beta cell function; HOMA-IR, HOMA for insulin resistance; LDL, low-density lipoprotein; PGC, Psychiatric Genomics Consortium; RGC, ReproGen Consortium; UKB, UK Biobank.

We compared the early-onset AN and typical-onset AN genetic correlations (Table S9 in Supplement 2). FDR-significant differences emerged for reproductive and anthropometric traits (Figure 1). Early-onset AN evidenced genetic overlap with younger age at menarche, whereas typical-onset AN did not. Early-onset AN did not genetically overlap with anthropometric traits, whereas typical-onset AN showed negative correlations.

### GRS_age of onset_, GRS_early-onset AN_, and GRS_AN_ as Predictors of Age of Onset of AN

**Figure 2:**
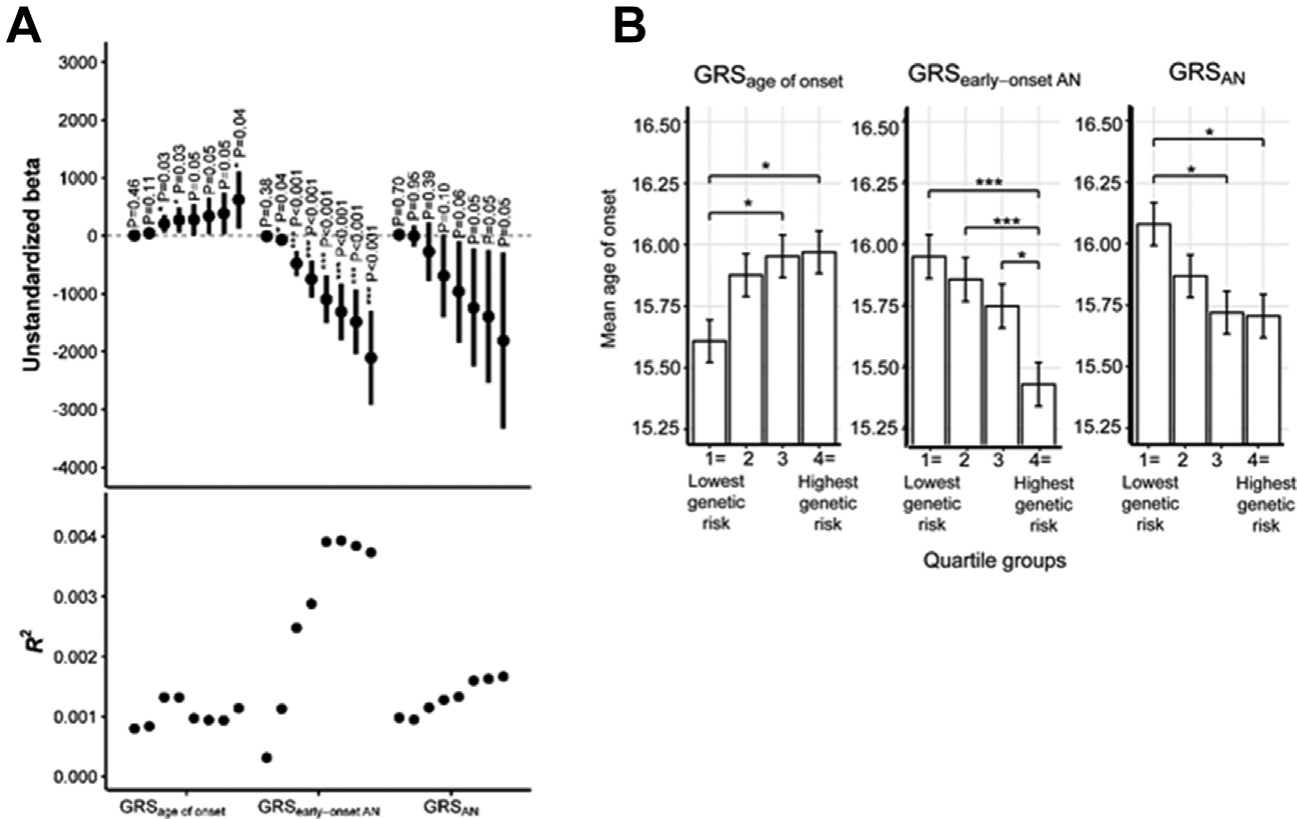
GRS_age of onset_ and GRS_early-onset AN_ significantly predict age of onset of AN. The *p* values are false discovery rate–corrected for multiple testing. ∗*p* < .05; ∗∗∗*p* < .001. **(A)** Unstandardized βs represent the average increase in age of onset (years) per 1-unit increase in GRS. The error bars are 95% confidence intervals. The eight data points in each field, from left to right, represent ascending *p* value selection thresholds (*p*_T_ < .001, *p*_T_ < .01, *p*_T_ < .1, *p*_T_ < .2, *p*_T_ < .3, *p*_T_ < .4, *p*_T_ < .5, *p*_T_ < 1) for inclusion of SNPs from the GWAS into the GRS (i.e., *p*_T_ < 1 means that all SNPs were included in score calculation). Table S10 in Supplement 2 reports descriptive statistics for GRS. **(B)** Marginal means and standard errors are plotted. The tests of significant difference are from fixed-effects inverse-variance weighted meta-analyses of mean difference in age of onset by GRS quartile. The data include 13 cohorts (*n* = 9335) for GRS_age of onset_ and GRS_AN_ and 5 cohorts (*n* = 8267) for GRS_early-onset AN_. AN, anorexia nervosa; GRS, genetic risk score; GRS_AAO_, GRS computed from the within-case age of onset GWAS; GRS_AN_, GRS computed from the case-control AN GWAS (2); GRS_early−onset AN_, GRS computed from the case-control early-onset AN GWAS; GWAS, genome-wide association study; SNP, single nucleotide polymorphism.

GRS analyses supported a common genetic basis of age of onset (Figure 2). Higher GRS_age of onset_ significantly predicted higher age of onset at three *p*_T_s, and GRS_early-onset AN_ significantly predicted a younger age of onset at seven *p*_T_s. GRS_AN_ did not significantly predict age of onset. The GRSs explained a small amount of phenotypic variance (*R*^2^) in age of onset (Figure 2). The highest *R*^2^s across *p*_T_s were 0.13% for GRS_age of onset_ (at *p*_T_ < .1), 0.39% for GRS_early-onset AN_ (*p*_T_ < .4), and 0.17% for GRS_AN_ (*p*_T_ < 1). Cochrane *Q*s for the meta-analyses in Figure 2 (24 total) were nonsignificant (*p* < .05), indicating that despite methodological differences across cohorts (i.e., age of onset phenotyping, recruitment, and sampling), heterogeneity was not evident. The forest plots in Figure S4 in Supplement 1 depict β estimates across the cohorts; results are shown for the best-performing *p*_T_s for illustration. We also observed significant associations between GRS quartile groups and age of onset (Supplemental Results in Supplement 1; Figure 2). Descriptive information for the GRS leave-one-cohort-out analyses is provided in Table S10 in Supplement 2.

### GRS_age at menarche_ as a Predictor of Age of Onset of AN, Early-Onset AN, Typical-Onset AN, and AN Risk

Per aim 3, we investigated the associations between GRS_age at menarche_ and age of onset (13 cohorts, 9335 cases), early-onset AN (5 cohorts, 1269 cases and 25,042 controls), typical-onset AN (5 cohorts, 6998 cases and 25,042 controls), and AN risk (13 cohorts, 11,632 cases and 31,981 controls). GRS_age at menarche_ significantly predicted age of onset of AN and early-onset AN at all *p*_T_s (Table 1). For instance, a 1 SD decrease in GRS_age at menarche_ was associated with an age of onset decrease of 0.21 years (95% CI −0.31 to −0.12; at *p*_T_ < .1) and a 20% higher odds of early-onset AN (95% CI 1.13 to 1.27; at *p*_T_ < 1).

**Table 1:** Association Between GRS_age at menarche_ and Age at Onset of AN, Early-Onset AN, Typical-Onset AN, and AN Risk

*p*_T_	Age of Onset of AN, 9335 Cases				Early-Onset AN, 1269 Cases, 25,042 Controls				Typical-Onset AN, 6998 Cases, 25,042 Controls				AN Risk, 11,632 Cases, 31,981 Controls			
	Estimates (95% CI)	*p* Value	*Q*	*R*^2^	Estimates (95% CI)	*p* Value	*Q*	*R*^2^	Estimates (95% CI)	*p* Value	*Q*	*R*^2^	Estimates (95% CI)	*p* Value	*Q*	*R*^2^
1	−0.19 (−0.28 to −0.10)	2.72 × 10^−5^^a^	23.92^a^	0.42%	1.20 (1.13 to 1.27)	4.13 × 10^−9^^a^	2.35	0.30%	0.99 (0.96 to 1.01)	.33	9.56	0.02%	1.02 (0.99 to 1.04)	.21	19.96	0.03%
.5	−0.19 (−0.28 to −0.10)	2.48 × 10^−5^^a^	22.63^a^	0.42%	1.20 (1.13 to 1.27)	4.81 × 10^−9^^a^	2.77	0.31%	0.98 (0.96 to 1.01)	.30	9.58^a^	0.02%	1.01 (0.99 to 1.04)	.23	19.78	0.03%
.4	−0.20 (−0.29 to −0.11)	1.52 × 10^−5^^a^	23.45^a^	0.44%	1.19 (1.12 to 1.27)	8.94 × 10^−9^^a^	2.96	0.30%	0.98 (0.96 to 1.01)	.29	8.79	0.02%	1.01 (0.99 to 1.04)	.24	19.64	0.03%
.3	−0.21 (−0.30 to −0.12)	8.00 × 10^−6^^a^	23.53^a^	0.46%	1.20 (1.13 to 1.27)	5.30 × 10^−9^^a^	2.32	0.30%	0.98 (0.95 to 1.01)	.16	8.01	0.02%	1.01 (0.99 to 1.04)	.41	18.72	0.03%
.2	−0.21 (−0.30 to −0.12)	8.59 × 10^−6^^a^	22.96^a^	0.46%	1.19 (1.12 to 1.26)	2.24 × 10^−8^^a^	2.47	0.27%	0.98 (0.95 to 1.01)	.16	9.37	0.02%	1.01 (0.99 to 1.04)	.39	20.34	0.03%
.1	−0.21 (−0.31 to −0.12)	6.13 × 10^−6^^a^	28.06^a^	0.51%	1.19 (1.12 to 1.27)	2.43 × 10^−8^^a^	3.74	0.29%	0.98 (0.95 to 1.00)	.10	10.50	0.02%	1.01 (0.99 to 1.04)	.34	20.94	0.03%
.01	−0.21 (−0.29 to −0.12)	7.24 × 10^−6^^a^	27.77^a^	0.52%	1.16 (1.09 to 1.23)	1.22 × 10^−6^^a^	2.03	0.23%	0.98 (0.95 to 1.00)	.09	5.73	0.02%	1.01 (0.99 to 1.04)	.33	9.95	0.02%
.001	−0.18 (−0.27 to −0.09)	4.96 × 10^−5^^a^	16.30	0.35%	1.13 (1.06 to 1.19)	8.00 × 10^−5^^a^	2.44	0.15%	0.97 (0.94 to 1.00)	.04	4.68	0.02%	1.01 (0.99 to 1.03)	.43	12.39	0.02%
1 × 10^−5^	−0.15 (−0.23 to −0.06)	1.12 × 10^−3^^a^	14.32	0.28%	1.10 (1.03 to 1.16)	2.57 × 10^−3^^a^	8.93	0.18%	0.96 (0.93 to 0.99)	.01	2.83	0.02%	1.00 (0.98 to 1.03)	.91	15.25	0.02%
5 × 10^−8^	−0.12 (−0.20 to −0.03)	.01^a^	10.69	0.19%	1.11 (1.05 to 1.18)	6.17 × 10^−4^^a^	4.16	0.14%	0.97 (0.94 to 1.00)	.05	1.40	0.01%	1.00 (0.98 to 1.03)	.73	8.92	0.02%

Estimates are β for age of onset of AN, and odds ratio for AN, early-onset AN, and typical-onset AN, and for interpretation purposes, reflect the effect of a standard deviation decrease in GRS_age at menarche_ (from the mean). *R*^2^ estimates for early-onset AN, typical-onset AN, and AN risk are liability-scale estimates.

AN, anorexia nervosa; GRS, genetic risk score; *p*_T_, the *p* value threshold for single nucleotide polymorphism selection for each GRS score.

a False discovery rate–adjusted *p* < .05.

### Age at Menarche as a Causal Risk Factor for Age of Onset of AN, Early-Onset AN, Typical-Onset AN, and AN Risk

Mendelian randomization provided evidence consistent with a causal link between younger age at menarche and early-onset AN (β = −0.21, SE = 0.09, *p* = .02) (Table 2). For each 1-year decrease in age at menarche below the mean (in the observed range), the odds of early-onset AN increased by 23% (95% CI 3% to 48%). Genetically determined age at menarche did not significantly predict age of onset of AN—although the mostly positive, wide CI precludes definitive interpretation (β = 0.23, 95% CI −0.02 to 0.48, *p* = .08)—and did not predict typical-onset AN (β = 0.06, 95% CI −0.04 to 0.16, *p* = .26) or AN risk (β = −0.04, 95% CI −0.12 to 0.04, *p* = .27). In the opposite direction, AN risk was not a causal risk factor for age at menarche (β = 0.03, 95% CI −0.05 to 0.11, *p* = .50). Given the lack of SNP instruments from the age of onset, early-onset, and typical-onset AN GWASs, their causal effects on age at menarche could not be investigated. Sensitivity analyses complemented the findings of the inverse-variance weighted analyses (Supplement 1).

**Table 2:** MR Analyses Testing the Causal Associations Between Age at Menarche and Age of Onset of AN, Early-Onset AN, Typical-Onset AN, and AN Risk

Analysis	Age at Menarche→Age of Onset of AN	Age at Menarche→Early-Onset AN	Age at Menarche→Typical-Onset AN	Age at Menarche→AN Risk	AN Risk→Age at Menarche
IVW MR, β (SE)	0.23 (0.13)	−0.21 (0.09)^a^	0.06 (0.05)	−0.04 (0.04)	0.03 (0.04)
Sensitivity Analyses					
Heterogeneity, *Q*	238.38	212.28	295.16^b^	395.54^b^	3.39
Egger regression, β (SE)	0.29 (0.38)	−0.20 (0.25)	0.17 (0.14)	0.13 (0.11)	0.41 (0.35)
Egger regression, intercept (SE)	−0.002 (0.02)	−0.0003 (0.01)	−0.005 (0.01)	−0.01 (0.004)	−0.03 (0.03)
Weighted median estimate, β (SE)	0.21 (0.20)	−0.09 (0.15)	0.07 (0.07)	0.0002 (0.05)	0.04 (0.04)
MR-PRESSO global	240.42	214.14	297.76^b^	399.03^b^	–
Steiger	Direction: true^a^	Direction: true^b^	Direction: true^b^	Direction: true^b^	Direction: true^b^
GSMR, β (SE)	0.07 (0.10)	−0.11 (0.07)	0.16 (0.03)^b^	0.03 (0.02)	0.03 (0.03)

β values are differences in the outcome per year increase in age at menarche for the analyses with menarche timing as the exposure. Number of single nucleotide polymorphism instruments in IVW, Egger, weighted median, and GSMR analyses: menarche→age of onset of AN = 208, menarche→early-onset AN = 210, age at menarche→typical-onset AN = 210, age at menarche→AN risk = 206, AN risk→age at menarche = 3.

AN, anorexia nervosa; GSMR, generalized summary data-based Mendelian randomization; IVW, inverse-variance weighted; MR-PRESSO, Mendelian randomization pleiotropy residual sum and outlier.

a *p* < .05.

b *p* < .001.

## Discussion

This study provides evidence for a genetic basis for age of onset of AN, which is conferred at least partly through common genetic variants. GRSs capturing the effects of alleles associated with AN age of onset and early-onset AN significantly predicted age of onset. Furthermore, results suggested that the genetic architecture underlying earlier puberty, represented by age at menarche, may bring about an earlier onset of AN.

The SNP-*h*^2^ of the subphenotype of early-onset AN was similar to what has been reported for psychiatric diagnoses including AN (SNP-*h*^2^s 0.10–0.26). The SNP-*h*^2^ of age of onset, a quantitative trait, was low (SNP-*h*^2^s 0.01–0.04). Other psychological and behavioral quantitative traits have been found to have low heritabilities (i.e., depressive symptoms, subjective well-being, cigarette smoking, extraversion: SNP-*h*^2^s 0.05–0.06) (16). Twin-based upper bound estimates of heritability (twin-*h*^2^) of age of onset are lacking but would help to contextualize these results, as would SNP-*h*^2^ estimates from large, homogeneous datasets (>5000) (45). The heritabilities of early-onset and typical-onset AN were similar. This paints a different picture to the Brainstorm Consortium’s report combining several psychiatric disorders, which suggested earlier age of onset of psychiatric illness as an indicator for higher heritability (16). The Brainstorm Consortium analysis was a broad brushstroke view that relied on rough single estimates of average age of onset from experts and did not use phenotypic data. Our approach is data based, but the estimates are preliminary, given that the discovery GWASs have small samples for psychiatric GWAS.

No loci reached genome-wide significance in the age of onset and early-onset AN GWASs. Precise epidemiological estimates of early-onset AN are lacking, but prevalence is low and less than the lifetime prevalence of 0.3% in adolescents and 0.9% in adults (1,28). This limits GWAS statistical power at the current sample sizes. The genetic correlation between early-onset AN and AN risk is stronger than the genetic correlation of AN risk with other traits (i.e., psychiatric, anthropometric, metabolic: *r*_g_s −0.36 to 0.45) (5). The genetic correlation between early-onset and typical-onset AN was high, although distinct patterns of genetic correlations with other traits were observed. Early-onset AN correlated with lower age at menarche, and typical-onset AN correlated negatively with anthropometric traits. Studying homogeneous clinical subphenotypes such as early-onset AN may yield novel insight into the etiology of AN but is reliant on large phenotyping collections. Preliminary findings from this study suggest that a reproductive biology–based etiology for some patients may be worthy of further exploration.

Two loci were associated with typical-onset AN, one highly multigenic locus containing many brain-expressed genes and associated with AN risk (5), and a single-gene locus encoding a centrosomal protein and associated with phenotypes genetically correlated with AN (5), such as body fat distribution (adjusted-for-BMI) (46), high-density lipoprotein cholesterol level (47), and unipolar depression (48). The typical-onset AN GWAS was statistically powered for the initial implication of variants, given that ORs of 1.12 to 1.28 are in the range of the larger effects that we would expect to see in AN.

An important finding in this study was that GRSs for age of onset and early-onset AN significantly predicted age of onset. This provides evidence for a common variant genetic basis for age of onset, although the clinical significance is unclear. The age of onset difference was approximately 6 months between the lowest and highest GRS_age of onset_ quartiles. Future studies characterizing the more extreme ends of the GRS distribution of age of onset (i.e., 5%) may see considerably greater differences in timing of AN onset. Irreversible growth stunting and long-term bone disease (49) are morbidities exacerbated by earlier onset and could be mitigated by risk prediction tools that predict not only illness risk but timing of onset.

The variance in age of onset accounted for by common genetic variants was low. The explanatory power of GRS is influenced by the underlying genetic architecture and heritability and sufficiently powered discovery GWASs. Even so, the ability to account for variance in psychiatric phenotypes has been limited. Schizophrenia, for example, has a large genetic component and a heritability estimate of 80% (50), but GRS has only been able to account for 7% of variation on the liability scale—the most of any psychiatric disorder—with this estimate increasing as GWAS power has increased (51). GRS broadly has proven useful in research applications—by predicting incidence, disease severity, and treatment response—and can yield benefit to personalized prevention and early intervention of disease even when accounting for low variance (52).

A prevailing difficulty in risk factor research for AN has been how to consider peripuberty as a puzzle piece. Early-onset AN was historically termed premenarchal AN (53), which failed to appreciate that the starvation emblematic of the disease arrests puberty (and omits males). The association between puberty timing and AN can be difficult to study because the low prevalence of AN renders prospective association studies impractical, and in treatment-seeking populations, patients typically have delays in help seeking. Genetic analyses circumvent some of these methodological difficulties. In this study, GRS for earlier age at menarche predicted early-onset AN and lower age of onset. Furthermore, there was evidence consistent with earlier age at menarche being a causal risk factor for early-onset AN. This could suggest a genetically distinct variant of AN, genetically linked to puberty that is predisposed to an earlier age of onset. Indeed, this aligns with previous hypotheses (54,55) that a subset of women with eating disorders may represent an ovarian hormone–sensitive phenotype. Furthermore, twin studies suggest that estrogen plays a role in genetic risk for disordered eating (17). Our results converge with clinical studies implicating early pubertal timing as a risk factor for eating disorders (17) and extend the literature by suggesting a shared or potentially causal genetic molecular basis.

Cases with early-onset AN did not show a genetic relationship with BMI or related anthropometric indices, in contrast to cases with typical-onset AN. Literature comparing premorbid BMI trajectory between these groups is lacking. Because metabolic factors in AN are a burgeoning area of study, this could be an interesting finding to follow up. Continued investigation of the subphenotype of early-onset AN may help to inform etiology and classification.

A limitation of this study is that the phenotyping of age of onset was not standardized across cohorts. The phenotype was age of onset of AN diagnosis in some cohorts and age of first symptoms in others. The reliance on retrospective recall of age of onset is also a limitation. The generalizability of the findings to non-Europeans and males is not known. Genetic effects may emerge earlier in males according to existing research (56). From the statistical power analyses, it seems reasonable to conclude that at this stage, the GWASs of age of onset and early-onset AN are underpowered to identify individual variants.

In conclusion, this study provides evidence that a genetic basis underlies AN age of onset and that reproductive biology may influence the early onset of AN. Larger, well-matched case-control samples with standardized age of onset data will help to further reveal the biological mechanisms that influence age of onset.

## Supplementary Material

Supplement 2

Key Resources Table

## References

[1] Hudson J.I., Hiripi E., Pope H.G., Kessler R.C. (2007). The prevalence and correlates of eating disorders in the National Comorbidity Survey Replication. Biol Psychiatry.

[2] Udo T., Grilo C.M. (2018). Prevalence and correlates of DSM-5-defined eating disorders in a nationally representative sample of U.S. adults. Biol Psychiatry.

[3] Zipfel S., Giel K.E., Bulik C.M., Hay P., Schmidt U. (2015). Anorexia nervosa: Aetiology, assessment, and treatment. Lancet Psychiatry.

[4] Yilmaz Z., Hardaway J.A., Bulik C.M. (2015). Genetics and epigenetics of eating disorders. Adv Genomics Genet.

[5] Watson H.J., Yilmaz Z., Thornton L.M., Hübel C., Coleman J.R.I., Gaspar H.A. (2019). Genome-wide association study identifies eight risk loci and implicates metabo-psychiatric origins for anorexia nervosa. Nat Genet.

[6] Javaras K.N., Runfola C.D., Thornton L.M., Agerbo E., Birgegård A., Norring C. (2015). Sex- and age-specific incidence of healthcare-register-recorded eating disorders in the complete Swedish 1979–2001 birth cohort. Int J Eat Disord.

[7] Favaro A., Busetto P., Collantoni E., Santonastaso P., de Girolamo G., McGorry P.D., Sartorius N. (2019). Age of Onset of Mental Disorders: Etiopathogenetic and Treatment Implications.

[8] Kipman A., Bruins-Slot L., Boni C., Hanoun N., Adès J., Blot P. (2002). 5-HT(2A) gene promoter polymorphism as a modifying rather than a vulnerability factor in anorexia nervosa. Eur Psychiatry.

[9] Plana M.T., Torres T., Rodríguez N., Boloc D., Gassó P., Moreno E. (2019). Genetic variability in the serotoninergic system and age of onset in anorexia nervosa and obsessive-compulsive disorder. Psychiatry Res.

[10] Dardennes R.M., Zizzari P., Tolle V., Foulon C., Kipman A., Romo L. (2007). Family trios analysis of common polymorphisms in the obestatin/ghrelin, BDNF and AGRP genes in patients with anorexia nervosa: Association with subtype, body-mass index, severity and age of onset. Psychoneuroendocrinology.

[11] Ribasés M., Gratacòs M., Fernández-Aranda F., Bellodi L., Boni C., Anderluh M. (2004). Association of BDNF with anorexia, bulimia and age of onset of weight loss in six European populations. Hum Mol Genet.

[12] Ferentinos P., Koukounari A., Power R., Rivera M., Uher R., Craddock N. (2015). Familiality and SNP heritability of age at onset and episodicity in major depressive disorder. Psychol Med.

[13] Hilker R., Helenius D., Fagerlund B., Skytthe A., Christensen K., Werge T.M. (2017). Is an early age at illness onset in schizophrenia associated with increased genetic susceptibility? Analysis of data from the Nationwide Danish Twin Register. EBiomedicine.

[14] Lin P.I., McInnis M.G., Potash J.B., Willour V., MacKinnon D.F., DePaulo J.R., Zandi P.P. (2006). Clinical correlates and familial aggregation of age at onset in bipolar disorder. Am J Psychiatry.

[15] Svensson A.C., Lichtenstein P., Sandin S., Öberg S., Sullivan P.F., Hultman C.M. (2012). Familial aggregation of schizophrenia: The moderating effect of age at onset, parental immigration, paternal age and season of birth. Scand J Public Health.

[16] Anttila V., Bulik-Sullivan B., Finucane H.K., Walters R.K., Bras J., Brainstorm Consortium (2018). Analysis of shared heritability in common disorders of the brain. Science.

[17] Klump K.L. (2013). Puberty as a critical risk period for eating disorders: A review of human and animal studies. Horm Behav.

[18] Mullins N., Lewis C.M. (2017). Genetics of depression: Progress at last. Curr Psychiatry Rep.

[19] Loeb K.L., Brown M., Goldstein M.M., Le Grange D., Lock J. (2011). Eating Disorders in Children and Adolescents: A Clinical Handbook.

[20] Peebles R., Wilson J.L., Lock J.D. (2006). How do children with eating disorders differ from adolescents with eating disorders at initial evaluation?. J Adolesc Health.

[21] Walker T., Watson H.J., Leach D.J., McCormack J., Tobias K., Hamilton M.J., Forbes D.A. (2014). Comparative study of children and adolescents referred for eating disorder treatment at a specialist tertiary setting. Int J Eat Disord.

[22] Steinhausen H.C. (2009). Outcome of eating disorders. Child Adolesc Psychiatr Clin N Am.

[23] Baker J.H., Thornton L.M., Bulik C.M., Kendler K.S., Lichtenstein P. (2012). Shared genetic effects between age at menarche and disordered eating. J Adolesc Health.

[24] Kirk K.M., Martin F.C., Mao A., Parker R., Maguire S., Thornton L.M. (2017). The Anorexia Nervosa Genetics Initiative: Study description and sample characteristics of the Australian and New Zealand arm. Aust N Z J Psychiatry.

[25] Thornton L.M., Munn-Chernoff M.A., Baker J.H., Juréus A., Parker R., Henders A.K. (2018). The Anorexia Nervosa Genetics Initiative (ANGI): Overview and methods. Contemp Clin Trials.

[26] Lam M., Awasthi S., Watson H.J., Goldstein J., Panagiotaropoulou G., Trubetskoy V. (2020). RICOPILI: Rapid Imputation for COnsortias PIpeLIne. Bioinformatics.

[27] Abecasis G.R., Auton A., Brooks L.D., DePristo M.A., Durbin R.M., 1000 Genomes Project Consortium (2012). An integrated map of genetic variation from 1,092 human genomes. Nature.

[28] Swanson S.A., Crow S.J., Le Grange D., Swendsen J., Merikangas K.R. (2011). Prevalence and correlates of eating disorders in adolescents. Results from the National Comorbidity Survey Replication Adolescent Supplement. Arch Gen Psychiatry.

[29] Gauderman W.J., Morrison J.M. (2009). Quanto 1.2.4: A computer program for power and sample size calculations for genetic epidemiology studies. https://pphs.usc.edu/download-quanto/.

[30] Finucane H.K., Bulik-Sullivan B., Gusev A., Trynka G., Reshef Y., Loh P.R. (2015). Partitioning heritability by functional annotation using genome-wide association summary statistics. Nat Genet.

[31] Yang J., Lee S.H., Goddard M.E., Visscher P.M. (2011). GCTA: A tool for genome-wide complex trait analysis. Am J Hum Genet.

[32] Visscher P.M., Hemani G., Vinkhuyzen A.A., Chen G.B., Lee S.H., Wray N.R. (2014). Statistical power to detect genetic (co)variance of complex traits using SNP data in unrelated samples. PLoS Genet.

[33] Zheng J., Erzurumluoglu A.M., Elsworth B.L., Kemp J.P., Howe L., Haycock P.C. (2017). LD Hub: A centralized database and web interface to perform LD score regression that maximizes the potential of summary level GWAS data for SNP heritability and genetic correlation analysis. Bioinformatics.

[34] Duncan L., Yilmaz Z., Gaspar H., Walters R., Goldstein J., Anttila V. (2017). Significant locus and metabolic genetic correlations revealed in genome-wide association study of anorexia nervosa. Am J Psychiatry.

[35] Watanabe K., Taskesen E., van Bochoven A., Posthuma D. (2017). Functional mapping and annotation of genetic associations with FUMA. Nat Commun.

[36] Choi S.W., O’Reilly P.F. (2019). PRSice-2: Polygenic risk score software for biobank-scale data. GigaScience.

[37] Benjamini Y., Hochberg Y. (1995). Controlling the false discovery rate: A practical and powerful approach to multiple testing. J R Stat Soc B.

[38] Day F.R., Thompson D.J., Helgason H., Chasman D.I., Finucane H., Sulem P. (2017). Genomic analyses identify hundreds of variants associated with age at menarche and support a role for puberty timing in cancer risk. Nat Genet.

[39] Hemani G., Zheng J., Elsworth B., Wade K.H., Haberland V., Baird D. (2018). The MR-Base platform supports systematic causal inference across the human phenome. Elife.

[40] Burgess S. (2014). Sample size and power calculations in Mendelian randomization with a single instrumental variable and a binary outcome. Int J Epidemiol.

[41] Makino K., Umeda K., Uezu A., Hiragami Y., Sakamoto T., Ihn H., Nakanishi H. (2008). Identification and characterization of the novel centrosomal protein centlein. Biochem Biophys Res Commun.

[42] Buckley M.A., Woods N.T., Tyrer J.P., Mendoza-Fandiño G., Lawrenson K., Hazelett D.J. (2019). Functional analysis and fine mapping of the 9p22.2 ovarian cancer susceptibility locus. Cancer Res.

[43] Keski-Rahkonen A., Mustelin L. (2016). Epidemiology of eating disorders in Europe: Prevalence, incidence, comorbidity, course, consequences, and risk factors. Curr Opin Psychiatry.

[44] Micali N., Martini M.G., Thomas J.J., Eddy K.T., Kothari R., Russell E. (2017). Lifetime and 12-month prevalence of eating disorders amongst women in mid-life: A population-based study of diagnoses and risk factors. BMC Med.

[45] Tropf F.C., Lee S.H., Verweij R.M., Stulp G., van der Most P.J., de Vlaming R. (2017). Hidden heritability due to heterogeneity across seven populations. Nat Hum Behav.

[46] Pulit S.L., Stoneman C., Morris A.P., Wood A.R., Glastonbury C.A., Tyrrell J. (2019). Meta-analysis of genome-wide association studies for body fat distribution in 694 649 individuals of European ancestry. Hum Mol Genet.

[47] Klarin D., Damrauer S.M., Cho K., Sun Y.V., Teslovich T.M., Honerlaw J. (2018). Genetics of blood lipids among ∼300,000 multi-ethnic participants of the Million Veteran Program. Nat Genet.

[48] Howard D.M., Adams M.J., Clarke T.K., Hafferty J.D., Gibson J., Shirali M. (2019). Genome-wide meta-analysis of depression identifies 102 independent variants and highlights the importance of the prefrontal brain regions. Nat Neurosci.

[49] Dede A.D., Lyritis G.P., Tournis S. (2014). Bone disease in anorexia nervosa. Hormones.

[50] Sullivan P.F., Kendler K.S., Neale M.C. (2003). Schizophrenia as a complex trait: Evidence from a meta-analysis of twin studies. Arch Gen Psychiatry.

[51] Schizophrenia Working Group of the Psychiatric Genomics Consortium (2014). Biological insights from 108 schizophrenia-associated genetic loci. Nature.

[52] Torkamani A., Wineinger N.E., Topol E.J. (2018). The personal and clinical utility of polygenic risk scores. Nat Rev Genet.

[53] Cooper P.J., Watkins B., Bryant-Waugh R., Lask B. (2002). The nosological status of early onset anorexia nervosa. Psychol Med.

[54] Hardin S.L., Thornton L.M., Munn-Chernoff M.A., Baker J.H. (2020). Premenstrual symptoms as a marker of ovarian hormone sensitivity in eating disorders. Int J Eat Disord.

[55] Baker J.H., Girdler S.S., Bulik C.M. (2012). The role of reproductive hormones in the development and maintenance of eating disorders. Expert Rev Obstet Gynecol.

[56] Culbert K.M., Burt S.A., Klump K.L. (2017). Expanding the developmental boundaries of etiologic effects: The role of adrenarche in genetic influences on disordered eating in males. J Abnorm Psychol.

