# Supplementary material for: Common Genetic Variation and Age of Onset of Anorexia Nervosa
Source: Biol Psychiatry Glob Open Sci. 2021 Sep 20;2(4):368–78. doi: 10.1016/j.bpsgos.2021.09.001 (PMC9616394; doi:10.1016/j.bpsgos.2021.09.001)
Supplement: Supplement 1 [file mmc1.pdf]

Supplement 1

Common Genetic Variation and Age of Onset of Anorexia Nervosa

Table of Contents

	Page
**Supplementary Methods**	
Recruitment and phenotyping	1
Age of onset phenotype	1
Genome-wide association study (GWAS) quality control and imputation	3
Genetic correlation differences	4
$GRS_{age\ of\ onset}$, $GRS_{early\text{-}onset\ AN}$, and GRS_{AN} as predictors of age of onset of AN	5
$GRS_{age\ at\ menarche}$ as a predictor of age of onset of AN, early-onset AN, typical-onset AN, and AN risk	6
Causal associations between puberty timing and age of onset of AN, early-onset AN, typical-onset AN, and AN risk	6
**Supplementary Results**	
Specificity of the phenotypes	7
GWAS of early-onset AN	8
$GRS_{age\ of\ onset}$, $GRS_{early\text{-}onset\ AN}$, and GRS_{AN} as predictors of age of onset of AN	9
$GRS_{age\ at\ menarche}$ as a predictor of age of onset of AN, early-onset AN, typical-onset AN, and AN risk	9

Age at menarche as a causal risk factor for AN onset: Mendelian randomization analyses		10
**Supplementary Figures and Legends**		
Fig S1.	Density plot representing the age of onset (years) of AN (13 cohorts, 9,335 cases)	11
Fig S2.	Plots for the (a) within-case GWAS analysis of age of onset of AN (13 cohorts, 9,335 cases), (b) case-control GWAS analysis of early-onset AN (5 cohorts, 1,269 cases and 25,042 controls), and (c) case-control GWAS analysis of typical-onset AN (5 cohorts, 6,998 cases and 25,042 controls), specifically the (i) Q-Q plot, (ii) Manhattan plot, and (iii) region plots for significant loci	12
Fig S3.	Tissue expression analysis of typical-onset AN GWAS summary statistics	18
Fig S4.	Per-cohort tests of association between $GRS_{\text{age of onset}}$, $GRS_{\text{early-onset AN}}$, GRS_{AN} and age of onset of AN	20
**Supplementary Tables**		
Table S1.	Descriptive summary of the cohorts	†
Table S2.	Phenotyping of age of onset	
Table S3.	Number of cases and controls in the genome-wide association study (GWAS) analyses of age of onset of AN, early-onset AN, and typical-onset AN	
Table S4.	MAGMA gene-set analysis for the typical-onset AN GWAS	

Tables S5a, S5b, and S5c.	SNP signals within the 8 genome-wide significant loci in the Psychiatric Genomics Consortium AN risk GWAS (Watson et al., 2019)	
Table S6.	Genetic correlations between early-onset AN and 62 selected traits	
Table S7.	Genetic correlations between early-onset AN and 770 traits	
Table S8.	Genetic correlations between typical-onset AN and 770 traits	
Table S9.	Differences in genetic correlations between early-onset AN and typical-onset AN among 62 selected traits	
Table S10.	Descriptive information for the genetic risk score (GRS) leave-one-cohort-out analyses	
**Supplementary References**		22
**Supplementary Appendix**<br>Eating Disorders Working Group of the Psychiatric Genomics Consortium		25

†See supplemental Excel file

SUPPLEMENTARY METHODS

Recruitment and Phenotyping

Cases had lifetime AN and were recruited from clinic and hospital settings, and from the community using traditional and social media. The majority of participants came from the ANGI study (74%) [1], and the remainder from the Wellcome Trust Case-Control Consortium 3 and Genetics Consortium of Anorexia Nervosa studies (WTCCC3/GCAN, 15%) [8] and the Children's Hospital of Philadelphia and Price Foundation Collaborative Group studies (CHOP/PFCG, 11%) [2]. Diagnoses were made based on DSM-III-R AN, ICD-10 AN, or DSM-IV AN or broad AN criteria (i.e., all DSM-IV criteria with the exception of amenorrhea and equivalent to DSM-5 criteria), and were assigned using structured diagnostic interviews or from diagnostic algorithms based on DSM-5 derived from survey responses. The controls in the cohorts included in the present study were screened for the absence of eating disorders (N = 31,981).

Age of Onset Phenotype

Measurement

For ANGI participants, age of onset was the age at which the first AN symptom including low BMI, fasting, self-induced vomiting, laxative/diuretic/diet pill use, and exercise to control weight or shape, first appeared and was captured by retrospective self-report. Age of onset of AN

diagnosis was not available. For CHOP/PFCG participants, age of onset was the age of onset of the diagnosis of AN and was determined by clinical interviewers with the Structured Clinical Interview for DSM-IV (SCID) or the Eating Disorder Examination (EDE) and was 16.7 ± 3.6 years (mean ± s.d.) [3], in comparison with the age at first symptoms of 15.1 ± 3.1 years [2]. For WTCCC3/GCAN participants, phenotype data were made available for secondary analysis by consortium collaborators. Phenotyping was site-dependent and was predominantly the age of onset of the diagnosis of AN assessed by clinicians with structured or semi-structured clinical interview instruments. Further information is shown in Supplementary Table S2.

Age of onset was treated as both a continuous variable and, for subphenotyping purposes, as a binary variable indicating early-onset AN. We defined early-onset as AN diagnosis before 13 years of age (N = 1,370) and typical-onset as AN diagnosis 13 years and after (N = 7,965), in accordance with epidemiological literature that adolescence is when AN most commonly onsets and childhood-onset is uncommon [4, 5]. We excluded two individuals with an age of onset < 3 years from the dataset (range of onset age: 5-58 years after exclusions).

Reliability and Validity

Providing evidence for reliability and validity, earlier age of onset has been found to have a more deleterious effect on physical development indices including bone mineral density and height [6-8], as would be expected. Age of onset has been found to be comparable when defined by DSM-IV or DSM-5 criteria [9]. Unfortunately, the measurement of age of onset in studies is rarely described, so the convergence between onset defined by occurrence of first diagnosis or

symptom/s is not known. Measurement of age of onset by diagnosis with a standardized clinical interview is believed to be the most reliable and valid form of measurement [9], since researchers who measure onset of first symptom/s may choose different symptoms and the line between normative and clinical behaviors can be blurry (e.g., many adolescent girls and boys try restricting eating for weight loss; [10]). There is no consensus in the literature for a threshold for differentiating early- from typical- or late-onset AN, but most studies tend to choose a chronological age that takes into account peak onset from epidemiological data, or based upon upon general population data on age at menarche, since adolescence is a key period of onset. For mental health diagnoses generally, a reliance on retrospective self-report and few data on reliability and validity is a rule rather than an exception, because of the practical impossibility of carrying out large prospective longitudinal birth cohort studies for validation [11].

Genome-Wide Association Study (GWAS) Quality Control and Imputation

The present study is a secondary analysis of datafiles from the Psychiatric Genomics Consortium (PGC) parent study [12]. The individual-level genotype files of the parent study were used. The quality control and imputation of these files within the parent study were as follows. They had been quality controlled, starting with the default quality control settings in RICOPILI, the PGCs analytic pipeline for performing GWAS [13]. RICOPILI begins with a pre-filter SNP call rate of > 0.95 in order to process data from cases and controls genotyped on different platforms. Default sample filters are a call rate (cases/controls) ≥ 0.98, heterozygosity inbreeding coefficient ≤ 0.2 (cases/controls), and sex violations. Default SNP filters are a call rate ≥ 0.98, case-control missingness difference ≤ 0.02, no valid association *P* value (invariant), and violations of Hardy-

Weinberg equilibrium (in controls $P > 10^{-6}$, in cases $P > 10^{-10}$). Some cohorts had needed stricter thresholds to reduce bias. Ancestry outliers had been removed by plotting the first two principal components (PCs) in a principal components analysis (PCA) containing each cohort and five reference cohorts (1000 Genomes Phase 3 EUR, AFR, EAS, SAS, AMR)[14]. Samples had been restricted to European ancestry based on cohort ascertainment information and confirmation via PCA with reference cohorts. Samples with familial structure and/or cryptic relatedness, or duplicates, had been removed ($\hat{\pi} > 0.2$) during PCA. To the extent that national laws and regulations permitted, sample overlap across cohorts had been assessed by performing LD score bivariate regressions and estimating genetic covariance intercepts [15, 16]. The cohorts had been imputed to the 1000 Genomes Phase 3 reference using the RICOPILI pipeline and were not reimputed for the purpose of this secondary analysis. For further QC information, see Supplementary Tables 18 and 19 of the parent study [12].

Genetic Correlation Differences

Early-onset AN and typical-onset AN genetic correlations were compared by using a Fisher *r*-to-*z* transformation on the r_g estimates in order to transform the correlations to a normal sampling distribution such that a test of significance could be conducted on the difference in the *z* estimates. The false discovery rate (FDR) procedure was applied to correct for multiple testing [17]. This approach was used in the absence of a method for use with partially dependent r_gs due to overlapping controls.

$GRS_{age\ of\ onset}$, $GRS_{early\text{-}onset\ AN}$, and GRS_{AN} as Predictors of Age of Onset of AN

The approach to calculating GRS entails three steps: (1) obtain a beta weight for each SNP based on a GWAS of the phenotype in a discovery sample; (2) restrict the SNPs to those in the discovery GWAS that exceed particular *P* value thresholds (we used $P_T < 1.0$, 0.5, 0.4, 0.3, 0.2, 0.1, 0.01, 0.001); and (3) calculate a GRS for each individual in the independent target sample, by summing risk alleles from the pre-defined SNP set weighted by the beta from the discovery sample GWAS. GRS were calculated in PRSice-2 [18] for each individual using the leave-one-cohort-out method, such that each cohort in the original GWAS was iteratively made to serve as the target sample while the remaining cohorts combined formed the discovery sample. This process ensures that an individual's GRS (i.e., for age of onset of AN) is calculated completely independently from the phenotype that the GRS will be used to prospectively predict. R^2 was calculated by subtracting the R^2 from the full model (GRS + covariates) from the R^2 of the covariate-only model. Weighted fixed-effects meta-analysis was used to combine βs (with inverse-variance weighting) and R^2 (with sample size weighting) across cohorts. *P* values for β were corrected for multiple testing within each predictor set using the FDR procedure.

We also examined $GRS_{age\ of\ onset}$, $GRS_{early\text{-}onset\ AN}$, and GRS_{AN}, as ordinal predictors by splitting case groups within the cohorts by GRS quartiles so that the more extreme ends of the risk distributions could be compared (i.e., Q1 at lowest genetic risk, Q4 at highest genetic risk). This was considered informative given that GRS lack precision, currently, based on limitations in the GWAS discovery sample sizes, and the difficulty validating age of onset measurement. Very

large sample sizes are needed for accurately quantifying the effects of risk-increasing alleles on psychiatric traits.

$GRS_{age\ at\ menarche}$ as a Predictor of Age of Onset of AN, Early-Onset AN, Typical-Onset AN, and AN Risk

GRSs were calculated at P_{TS} 5×10^{-8}, 1×10^{-5}, 0.001, 0.01, 0.1, 0.2, 0.3, 0.4, 0.5, and 1 using PRSice-2 [18].. Linear regressions tested the associations between $GRS_{age\ at\ menarche}$ and age of onset of AN and logistic regressions tested the associations between $GRS_{age\ at\ menarche}$ and early-onset AN, typical-onset AN, and AN risk. R^2 was calculated by subtracting the R^2 from the full model (GRS + covariates) from the R^2 of the covariate-only model and where indicated Nagelkerke's R^2 on the observed scale was transformed to liability scale R^2 [19]. Weighted fixed-effects meta-analysis was used to combine effect estimates (with inverse-variance weighting) and R^2 (with sample size weighting) across cohorts. *P* values were corrected for multiple testing within each outcome set using the FDR procedure [17].

Causal Associations Between Puberty Timing and Age of Onset of AN, Early-onset AN, Typical-Onset AN, and AN Risk

Secondary to the main Mendelian randomization analysis, sensitivity analyses were carried out to assess the influence of horizontal pleiotropy, outliers, and reverse causality, including MR-Egger regression [20], weighted median estimator [21], Egger intercept test, Cochran *Q* heterogeneity statistic [22], MR-PRESSO [23], Steiger test of directionality [24], and generalized

summary data-based Mendelian randomization (GSMR) [25]. GSMR was carried out with GCTA-GSMR [25]. Egger regression and weighted median meta-analysis approaches are not as powerful as IVW so the direction and magnitude of the effects were considered instead of relying on *P* values.

SUPPLEMENTARY RESULTS

Specificity of the Phenotypes

With clinical data available for some AN samples, we considered whether other clinical features were confounding variables or "hitchhikers" such that age of onset of AN and early-onset AN represented other clinical traits of AN. We tested 90+ clinical variables collected among ANGI participants with the ED100k questionnaire resulting in an average 3,705 AN participants per test (*SD* = 1,977 participants per test). Based on the results below, the findings appear to have specificity for this phenotypes of interest and are not confounded by other clinical variables.

Age of Onset

We computed associations between continuous age of onset and the clinical variables, using correlation coefficients for continuous clinical variables and *t* tests for categorical clinical variables. We considered other variables to be confounding variables if they met a threshold of association of $r \geq 0.7$ (i.e., multicollinearity) or Cohen's $d \geq 0.5$, i.e., medium). Only three variables did; two were aspects of the phenotyping definition (i.e., age at first time of low weight or AN, and age at first fasting) and the other was "Before this time when you were at low weight

or had AN, had your periods started?" such that participants who responded "yes" were older when their AN started. In sum, no variables showed evidence of confounding the phenotype of interest of age of onset. This included traits such as subtype of AN (restricting or binge eating/purging), lifetime bulimia nervosa, and lowest BMI, which were not significantly associated (data not shown).

Early-Onset AN

We investigated the associations between the 90+ clinical variables and early-onset AN using *t* tests for continuous clinical variables (groups = early-onset and typical-onset) and χ^2 tests for categorical clinical variables. Cohen's $d > 0.5$ (i.e., medium) and phi > 0.1 (i.e., small) were interpreted as evidence of association. Six clinical variables were associated with early-onset AN, and all were related to the phenotyping definition: ages at first fasting, compulsive exercising, binge eating, low weight or AN, and self-induced vomiting, and whether menarche had occurred before AN.

GWAS of Early-Onset AN

One hit, which was evaluated as a false positive, was significant at the standard (non-Bonferroni-corrected) threshold of 5×10^{-8} (chr 5, rs181008825, OR = 2.29, 95% CI: 1.68, 3.13, $P = 1.99 \times 10^{-8}$).

$GRS_{age\ of\ onset}$, $GRS_{early\text{-}onset\ AN}$, and GRS_{AN} as Predictors of Age of Onset of AN

We also observed significant associations between GRS quartile groups and age of onset. The results are shown in Supplementary Figure S3 for the best-performing P_{TS} for illustration. Cases in Q4 of $GRS_{age\ of\ onset}$ had a significantly older age of onset than cases in Q1 (mean diff = 0.36 yrs, 95% CI: 0.12, 0.60, $P = 0.003$, FDR $P = 0.01$), cases in Q4 of GRS_{AN} had a significantly younger age of onset than cases in Q1 (mean diff = -0.36 yrs, 95% CI: -0.61, -0.12, $P = 0.004$, , FDR $P = 0.01$), and cases in Q4 of $GRS_{early\text{-}onset\ AN}$ had a significantly younger age of onset than cases in Q1 (mean diff = -0.52 yrs, 95% CI: -0.77, -0.27, $P = 4.02 \times 10^{-5}$, FDR $P = 0.0002$). There were further significant differences between the quartile groups, as shown in Figure 2. Cochran Q statistics for the meta-analyses of the comparisons conducted were predominantly (89%, i.e., 16/18 meta-analyses) non-significant ($Ps > 0.05$). The evidence for a dose-response association was considered statistically and visually, but neither supported nor refuted a dose-response association, as seen in Figure 2. Hence, in sum, the GRS analyses supported a common variant basis for AN age of onset.

$GRS_{age\ at\ menarche}$ as a Predictor of Age of Onset of AN, Early-Onset AN, Typical-Onset AN, and AN Risk

For the meta-analysis on the association between $GRS_{age\ at\ menarche}$ and AN risk, the full number of participants in each cohort were included in analyses (i.e., not just those individuals with age of onset data).

Age at Menarche as a Causal Risk Factor for AN Onset: Mendelian Randomization Analyses

We conducted sensitivity analyses to evaluate the robustness of the IVW results. The Mendelian randomization analysis is based on the assumption of no horizontal pleiotropy. For two of the outcomes, Cochran's Q tests ($P < 0.05$) and MR-PRESSO detected the presence of horizontal pleiotropy and heterogeneity (Table 2). The MR-PRESSO distortion test was used to compare the causal relationship before and after correction for pleiotropy. There was no evidence that the two causal estimates were significantly different after removing potentially pleiotropic SNPs. The Egger intercept provides an estimate for the directional bias due to horizontal pleiotropy, and was close to zero and not statistically significant for the five analyses, indicating a low risk of bias in the findings. The results from Egger regression, weighted median, and GSMR were similar in strength and direction to the IVW estimates. In contrast to IVW, for GSMR, genetically determined younger age at menarche was not significantly associated with early-onset AN but older age at menarche was significantly associated with typical-onset AN. Steiger's test supported the causal directions of the associations.

SUPPLEMENTARY FIGURES

Supplementary Figure S1. Density plot representing the age of onset (years) of AN (13 cohorts, 9,335 cases).

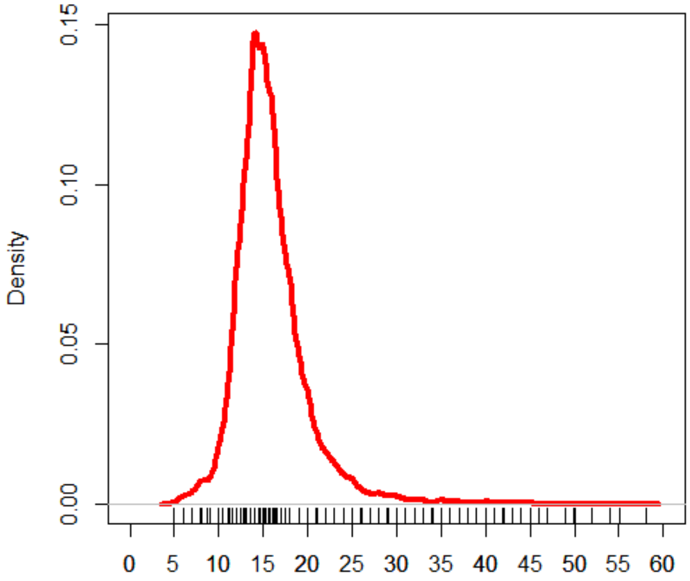

Supplementary Figure S2. Plots for the (a) within-case GWAS analysis of age of onset of AN (13 cohorts, 9,335 cases) and (b) case-control GWAS analysis of early-onset AN (5 cohorts, 1,269 cases and 25,042 controls), and (c) case-control GWAS analysis of typical-onset AN (cohorts, 6,998 cases and 25,042 controls), specifically the (i) Q-Q plot, (ii) Manhattan plot, and (iii) region plots for significant loci. Meta-analysis results are filtered to MAF > 0.01 and imputation INFO score > 0.7. Lambda was 1.01, LD intercept was 1.01 (s.e. = 0.01), and 9,027,579 genetic variants were tested in **a**, lambda was 1.05, LD intercept was 1.02 (s.e. = 0.01), and 9,078,137 genetic variants were tested in **b,** and lambda was 1.10, LD intercept was 1.01 (s.e. = 0.01), and 9,071,142 genetic variants were tested in **c**. (ii) The y-axis shows log-scaled *P* values. The red dotted line indicates nominal genome-wide significance (5×10^{-8}; although we applied a more stringent multiple-testing corrected genome-wide significance 1.7×10^{-8}). The light and dark blue colors in the Manhattan plots differentiate adjacent chromosomes.

a_i

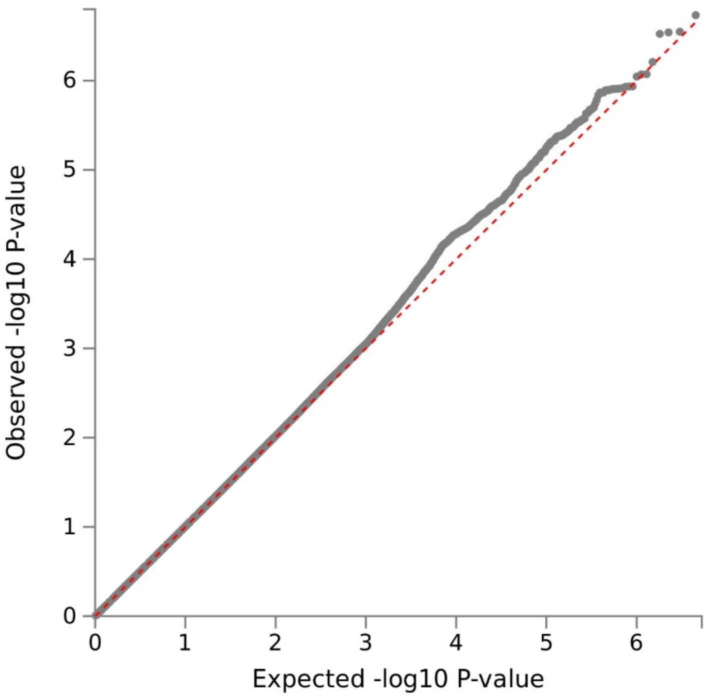
Observed -log10 P-value
Expected -log10 P-value
0
1
2
3
4
5
6

a_{ii}

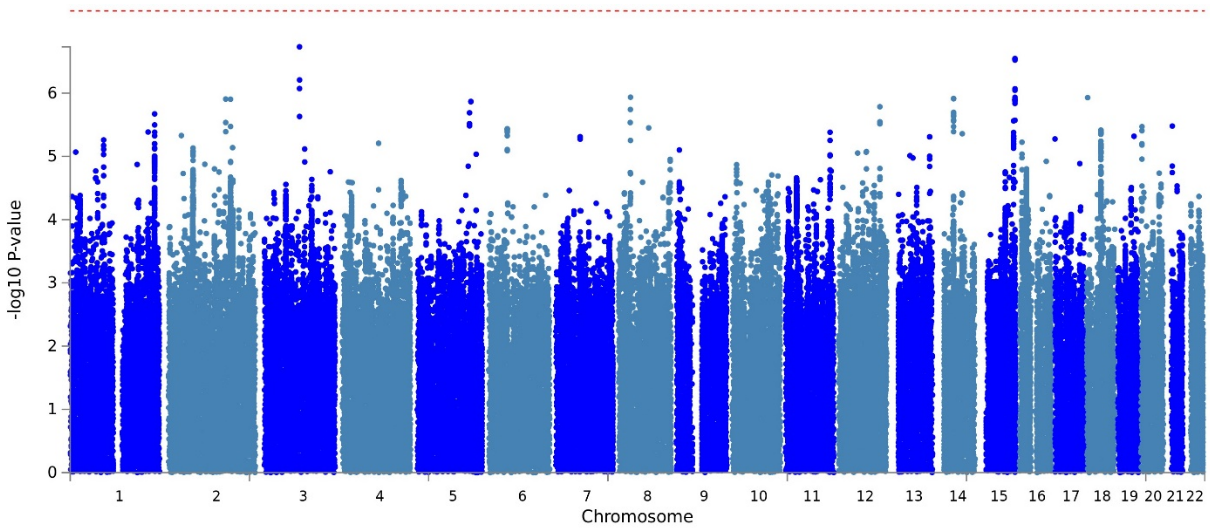
-log10 P-value
Chromosome
0
1
2
3
4
5
6
1
2
3
4
5
6
7
8
9
10
11
12
13
14
15
16
17
18
19
20
21
22

b$_i$

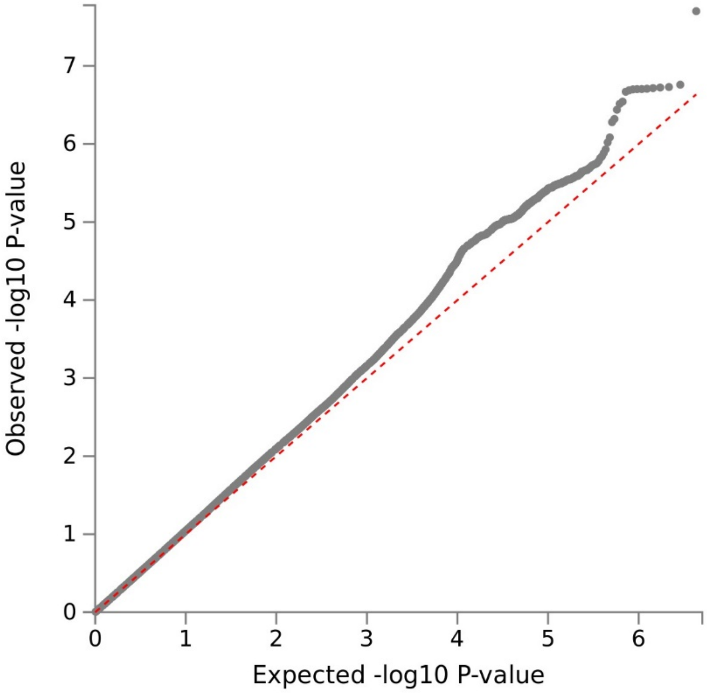
Observed -log10 P-value
Expected -log10 P-value
0
1
2
3
4
5
6
7

b$_{ii}$

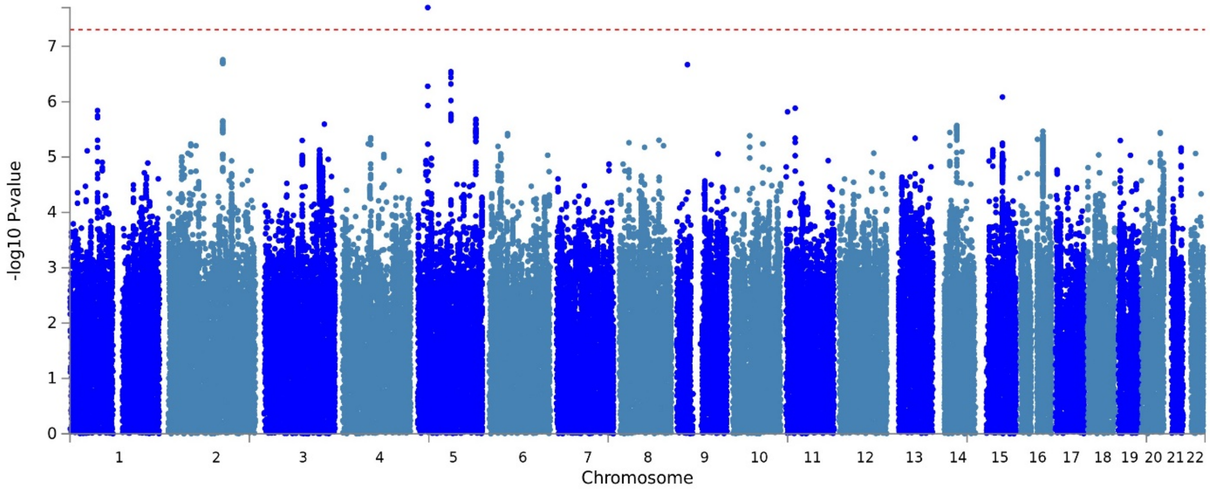
-log10 P-value
Chromosome
1
2
3
4
5
6
7
8
9
10
11
12
13
14
15
16
17
18
19
20
21
22

b_{iii}

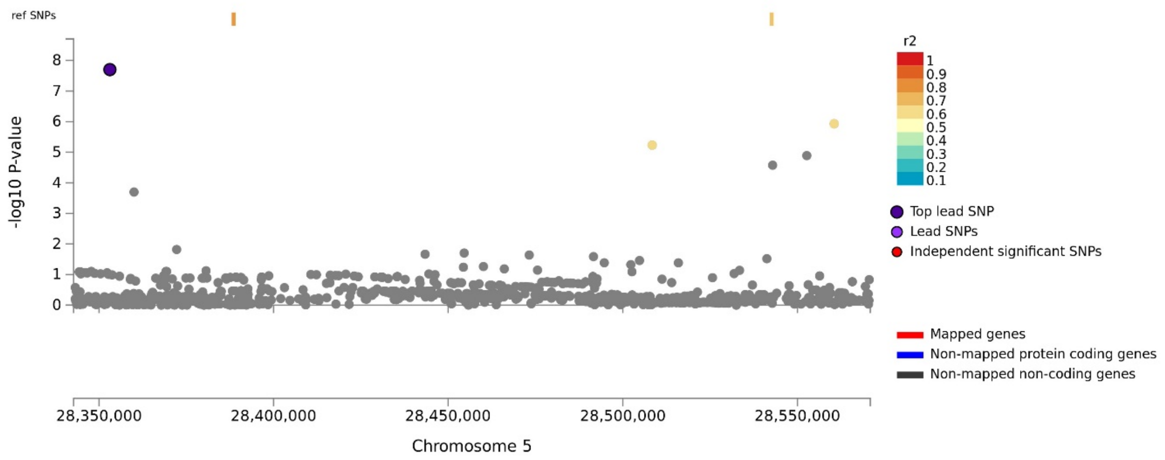
ref SNPs
-log10 P-value
Chromosome 5
28,350,000
28,400,000
28,450,000
28,500,000
28,550,000
r2
Top lead SNP
Lead SNPs
Independent significant SNPs
Mapped genes
Non-mapped protein coding genes
Non-mapped non-coding genes

c_i

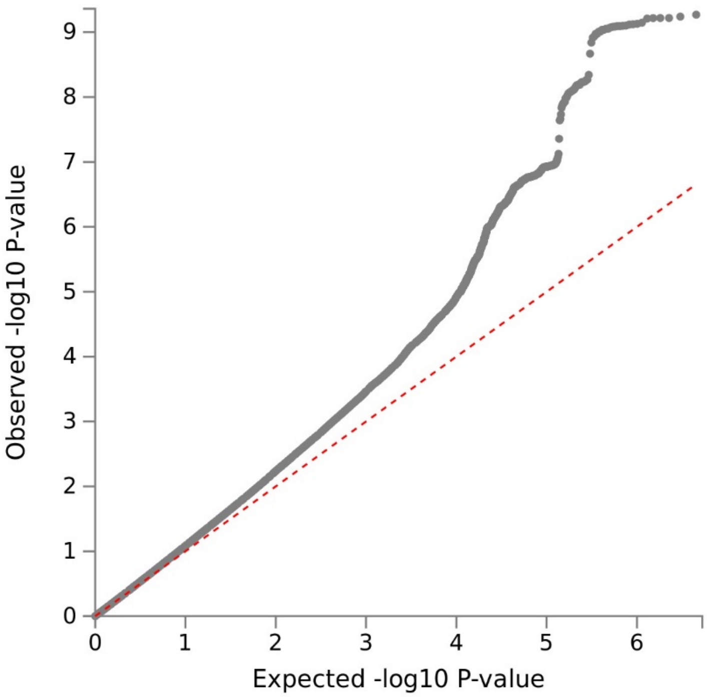
Observed -log10 P-value
Expected -log10 P-value

c$_{ii}$

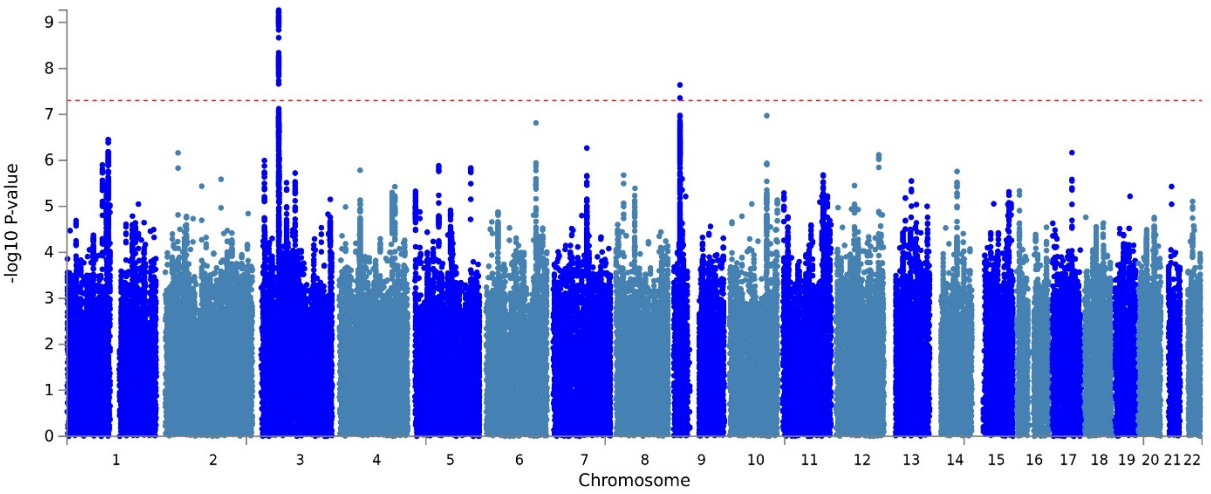
-log10 P-value
Chromosome
1
2
3
4
5
6
7
8
9
10
11
12
13
14
15
16
17
18
19
20
21
22

c$_{iii}$

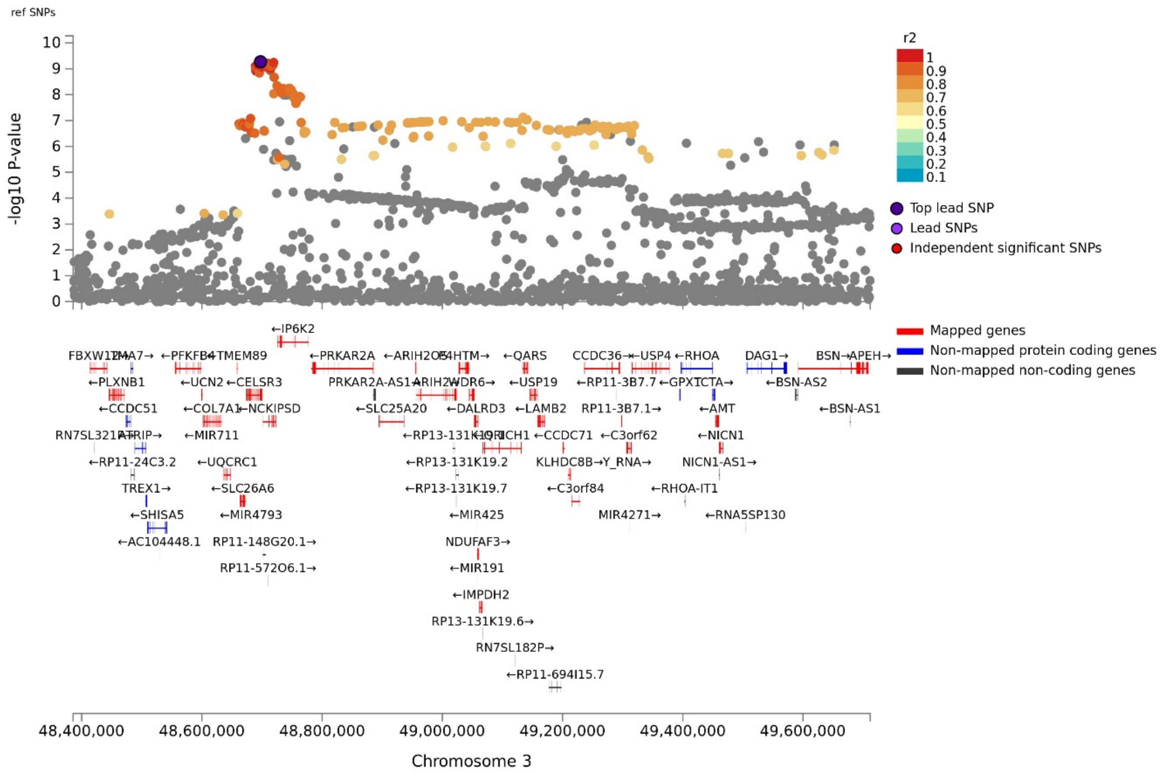
ref SNPs
-log10 P-value
r2
Top lead SNP
Lead SNPs
Independent significant SNPs
Mapped genes
Non-mapped protein coding genes
Non-mapped non-coding genes
Chromosome 3
48,400,000
48,600,000
48,800,000
49,000,000
49,200,000
49,400,000
49,600,000

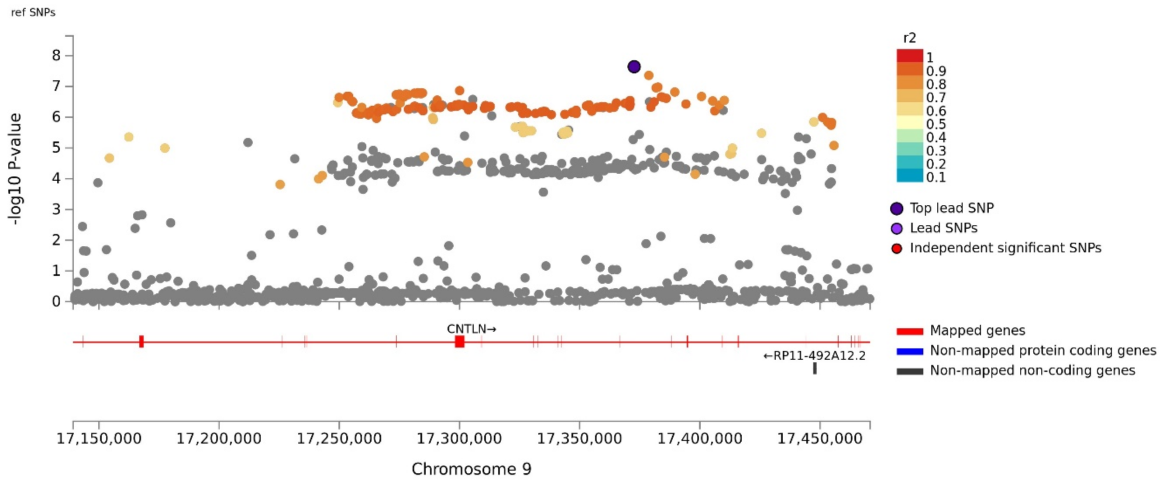
ref SNPs
-log10 P-value
r2
1
0.9
0.8
0.7
0.6
0.5
0.4
0.3
0.2
0.1
Top lead SNP
Lead SNPs
Independent significant SNPs
CNTLN→
←RP11-492A12.2
Mapped genes
Non-mapped protein coding genes
Non-mapped non-coding genes
17,150,000
17,200,000
17,250,000
17,300,000
17,350,000
17,400,000
17,450,000
Chromosome 9

Supplementary Figure S3. Tissue expression analysis of typical-onset AN GWAS summary statistics. MAGMA tissue expression analysis using gene expression per tissue based on GTEx RNA-seq data for (**a**) 30 general tissue types and (**b**) 53 specific tissue types. Significant enrichment is represented by a $-\log_{10} P > 2.8$ in **a** and > 3.03 in **b,** which takes multiple comparisons into account with a Bonferroni correction (i.e., 0.05/30 general tissue types and 0.05/53 specific tissue types).

a

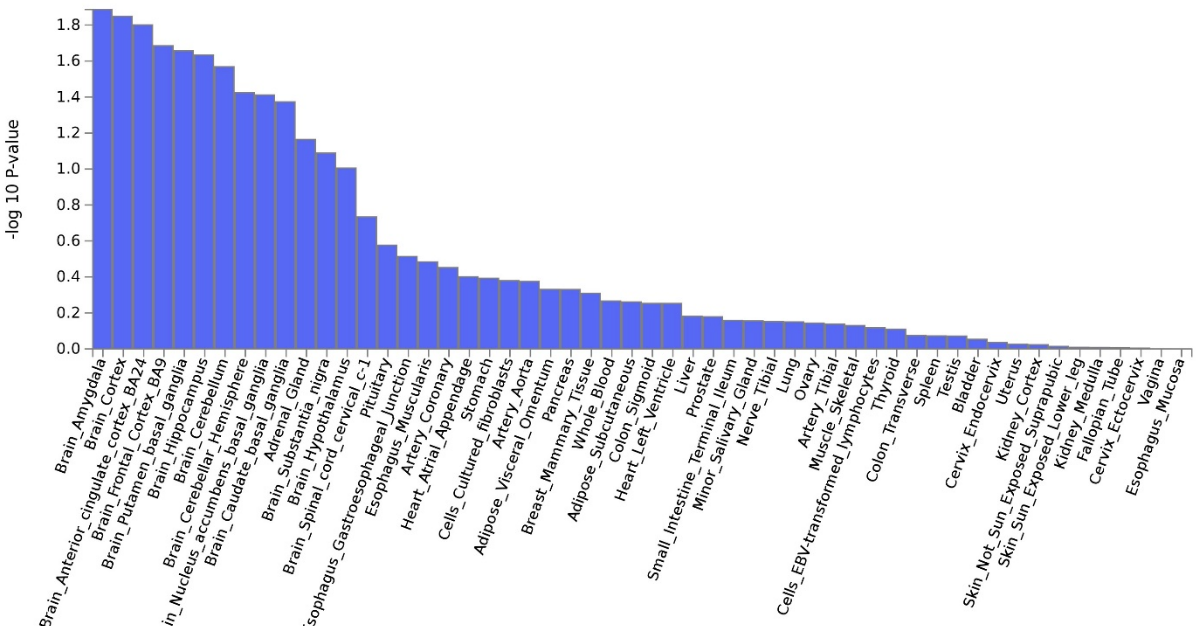

b

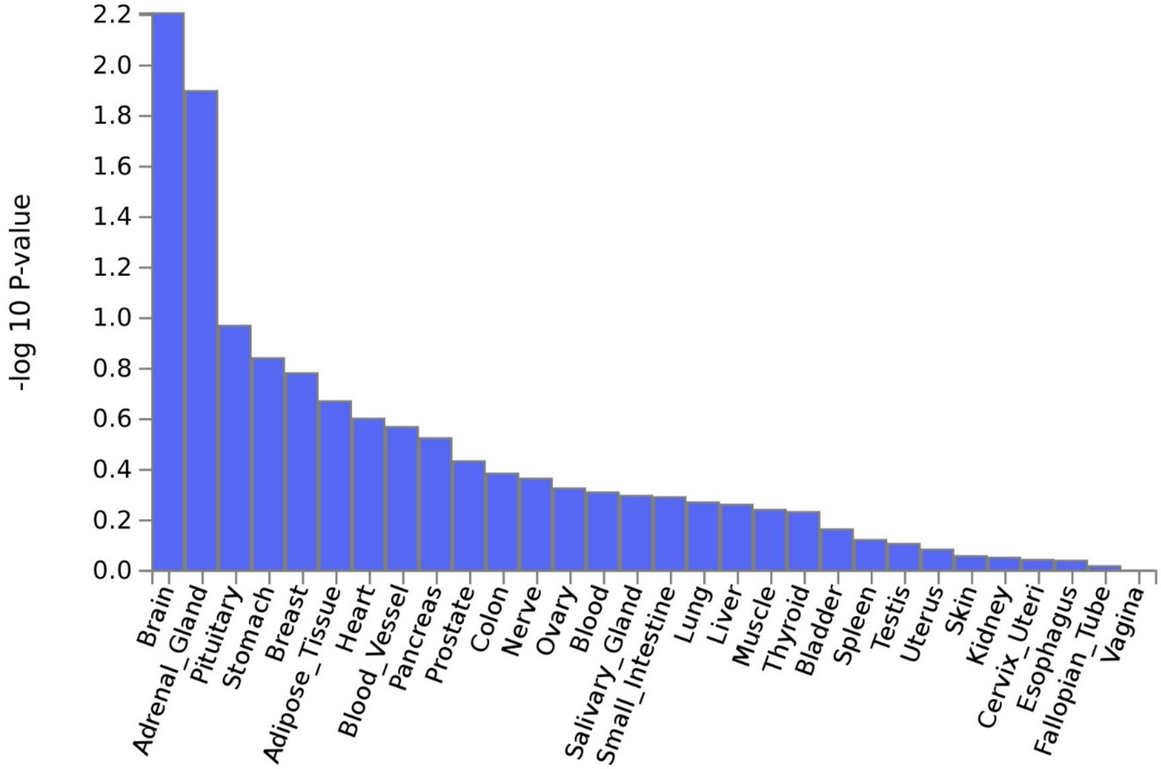
-log 10 P-value
2.2
2.0
1.8
1.6
1.4
1.2
1.0
0.8
0.6
0.4
0.2
0.0
Brain
Adrenal_Gland
Pituitary
Stomach
Breast
Adipose_Tissue
Heart
Blood_Vessel
Pancreas
Prostate
Colon
Nerve
Ovary
Blood
Salivary_Gland
Small_Intestine
Lung
Liver
Muscle
Thyroid
Bladder
Spleen
Testis
Uterus
Skin
Kidney
Cervix_Uteri
Esophagus
Fallopian_Tube
Vagina

Supplementary Figure S4. Per-cohort tests of association between $GRS_{age\ of\ onset}$, $GRS_{early\text{-}onset\ AN}$, GRS_{AN} and age of onset of AN. Depicted are the per-cohort results for the associations between GRSs calculated at the best-performing *P*-value thresholds for each GRS and age of onset of AN. The figures show the results for the predictors (**a**) $GRS_{age\ of\ onset}$ at $P_T < 0.1$ (**b**) GRS_{AN} at $P_T < 1$; and (**c**) $GRS_{early\text{-}onset\ AN}$ at $P_T < 0.3$. Unstandardized betas are reported as effect size estimates, interpreted as the average increase in age of onset (years) per 1-unit increase in GRS. The vertical reference line at 0 represents no effect. Study names are shown in uppercase and Psychiatrics Genomics Consortium (PGC) abbreviations are shown in lowercase for each cohort. The point estimate is shown in red and the 95% confidence interval (CI) is the horizontal blue line. $GRS_{age\ of\ onset}$ = GRS computed from the within-case age of onset GWAS. GRS_{AN} = GRS computed from the case-control AN GWAS [2]. $GRS_{early\text{-}onset\ AN}$ = GRS computed from the case-control early-onset AN GWAS. Supplementary Table S9 reports descriptive information for GRS analyses. In **a**, effects to the right of the zero-effect line indicate that as $GRS_{age\ of\ onset}$ increases, age of onset increases. In **b**, effects to the left of the zero-effect line indicate that as GRS_{AN} increases, age of onset decreases. In **c**, effects to the left of the zero-effect line indicate that as $GRS_{early\text{-}onset\ AN}$ increases, age of onset decreases. The summary effect and its 95% CI is the estimate from the fixed-effects inverse-variance weighted meta-analysis. The thickness of the horizontal line is a visual enhancement that represents larger studies with smaller confidence intervals and more weight in the meta-analysis.

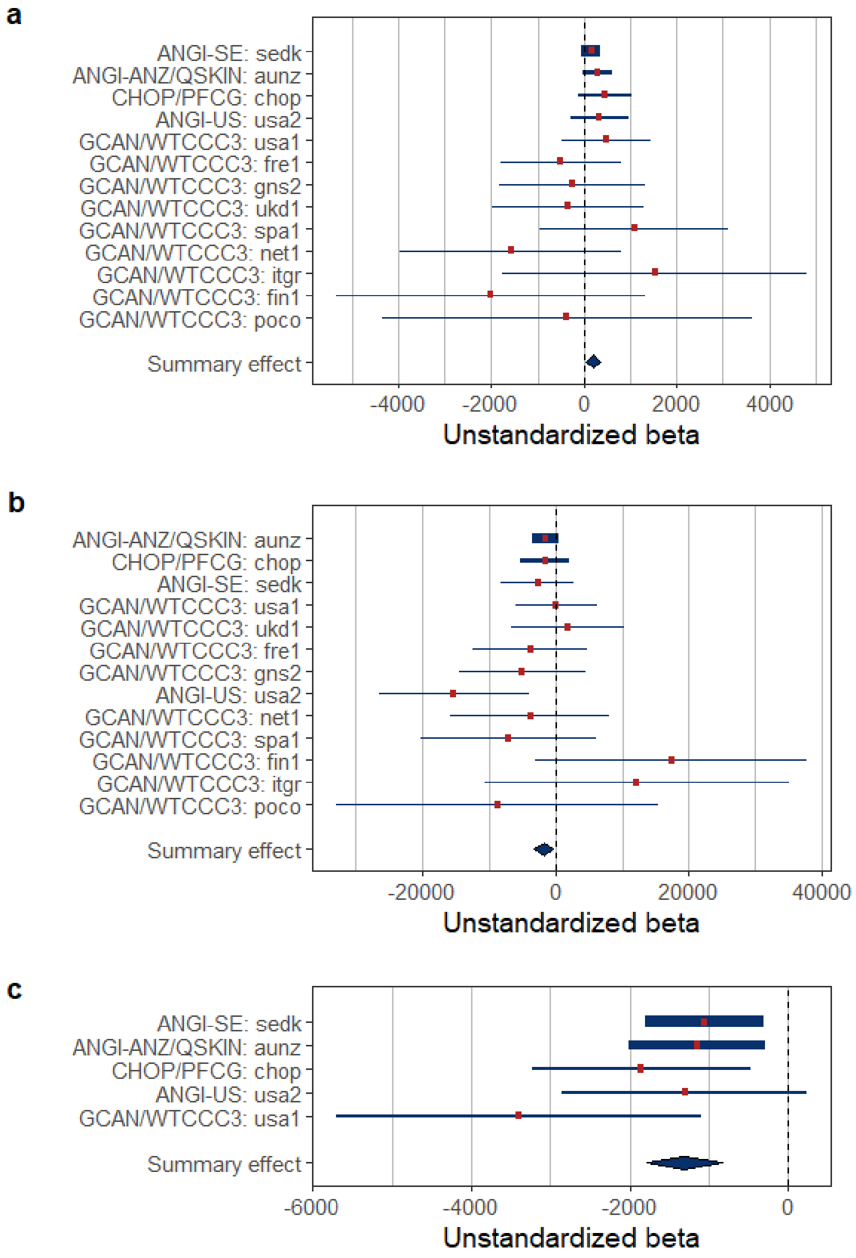
a
ANGI-SE: sedk
ANGI-ANZ/QSKIN: aunz
CHOP/PFCG: chop
ANGI-US: usa2
GCAN/WTCCC3: usa1
GCAN/WTCCC3: fre1
GCAN/WTCCC3: gns2
GCAN/WTCCC3: ukd1
GCAN/WTCCC3: spa1
GCAN/WTCCC3: net1
GCAN/WTCCC3: itgr
GCAN/WTCCC3: fin1
GCAN/WTCCC3: poco
Summary effect
-4000
-2000
0
2000
4000
Unstandardized beta
b
ANGI-ANZ/QSKIN: aunz
CHOP/PFCG: chop
ANGI-SE: sedk
GCAN/WTCCC3: usa1
GCAN/WTCCC3: ukd1
GCAN/WTCCC3: fre1
GCAN/WTCCC3: gns2
ANGI-US: usa2
GCAN/WTCCC3: net1
GCAN/WTCCC3: spa1
GCAN/WTCCC3: fin1
GCAN/WTCCC3: itgr
GCAN/WTCCC3: poco
Summary effect
-20000
0
20000
40000
Unstandardized beta
c
ANGI-SE: sedk
ANGI-ANZ/QSKIN: aunz
CHOP/PFCG: chop
ANGI-US: usa2
GCAN/WTCCC3: usa1
Summary effect
-6000
-4000
-2000
0
Unstandardized beta

SUPPLEMENTARY REFERENCES

1. Thornton, L.M., et al., *The Anorexia Nervosa Genetics Initiative (ANGI): Overview and methods.* Contemporary Clinical Trials, 2018. **74**: p. 61-69.
2. Wang, K., et al., *A genome-wide association study on common SNPs and rare CNVs in anorexia nervosa.* Molecular Psychiatry, 2011. **16**(9): p. 949-959.
3. Fichter, M.M., et al., *Structured Interview for Anorexic and Bulimic disorders for DSM-IV and ICD-10: Updated (third) revision.* International Journal of Eating Disorders, 1998. **24**(3): p. 227-249.
4. Hudson, J.I., et al., *The prevalence and correlates of eating disorders in the National Comorbidity Survey Replication.* Biological Psychiatry, 2007. **61**(3): p. 348-358.
5. Swanson, S.A., et al., *Prevalence and correlates of eating disorders in adolescents: Results from the National Comorbidity Survey Replication Adolescent Supplement.* Archives of General Psychiatry, 2011. **68**(7): p. 714-723.
6. Favaro, A., et al., *Association between low height and eating disorders: cause or effect?* International Journal of Eating Disorders, 2007. **40**(6): p. 549-553.
7. Maïmoun, L., et al., *Evidence of a link between resting energy expenditure and bone remodelling, glucose homeostasis and adipokine variations in adolescent girls with anorexia nervosa.* Osteoporos International, 2016. **27**(1): p. 135-146.
8. Joughin, N., et al., *Relative tallness in anorexia nervosa.* International Journal of Eating Disorders, 1992. **12**(2): p. 195-207.
9. Favaro, A., et al., *The age of onset of eating disorders*, in *Age of onset of mental disorders: Etiopathogenetic and treatment implications*, G. de Girolamo, P.D. McGorry,

and N. Sartorius, Editors. 2019, Springer International Publishing: Cham, Switzerland. p. 203-216.

10. Neumark-Sztainer, D., et al., *Dieting and disordered eating behaviors from adolescence to young adulthood: Findings from a 10-year longitudinal study.* Journal of the American Dietetic Association, 2011. **111**(7): p. 1004-1011.
11. Kessler, R.C., et al., *Age of onset of mental disorders: A review of recent literature.* Current Opinion in Psychiatry, 2007. **20**(4): p. 359-364.
12. Watson, H.J., et al., *Genome-wide association study identifies eight risk loci and implicates metabo-psychiatric origins for anorexia nervosa.* Nature Genetics, 2019. **51**(8): p. 1207-1214.
13. Lam, M., et al., *RICOPILI: Rapid Imputation for COnsortias PIpeLIne.* Bioinformatics, 2019. **36**(3): p. 930-933.
14. 1000 Genomes Project Consortium, et al., *An integrated map of genetic variation from 1,092 human genomes.* Nature, 2012. **491**(7422): p. 56-65.
15. Bulik-Sullivan, B.K., et al., *LD Score regression distinguishes confounding from polygenicity in genome-wide association studies.* Nat. Genet., 2015. **47**(3): p. 291-295.
16. Bulik-Sullivan, B., et al., *An atlas of genetic correlations across human diseases and traits.* Nat. Genet., 2015. **47**(11): p. 1236-1241.
17. Benjamini, Y. and Y. Hochberg, *Controlling the false discovery rate: A practical and powerful approach to multiple testing.* Journal of the Royal statistical society: series B (Methodological), 1995. **57**(1): p. 289-300.
18. Choi, S.W. and P.F. O'Reilly, *PRSice-2: Polygenic risk score software for biobank-scale data.* Gigascience, 2019. **8**(7): p. giz082.

19. Lee, S.H., et al., *A better coefficient of determination for genetic profile analysis.* Genetic Epidemiology, 2012. **36**(3): p. 214-224.

20. Bowden, J., G. Davey Smith, and S. Burgess, *Mendelian randomization with invalid instruments: Effect estimation and bias detection through Egger regression.* International Journal of Epidemiology, 2015. **44**(2): p. 512-525.

21. Bowden, J., et al., *Consistent estimation in Mendelian randomization with some invalid instruments using a weighted median estimator.* Genetic Epidemiology, 2016. **40**(4): p. 304-314.

22. Bowden, J., G. Hemani, and G. Davey Smith, *Invited commentary: Detecting individual and global horizontal pleiotropy in Mendelian randomization-a job for the humble heterogeneity statistic?* American Journal of Epidemiology, 2018. **187**(12): p. 2681-2685.

23. Verbanck, M., et al., *Detection of widespread horizontal pleiotropy in causal relationships inferred from Mendelian randomization between complex traits and diseases.* Nature Genetics, 2018. **50**(5): p. 693-698.

24. Hemani, G., K. Tilling, and G. Davey Smith, *Orienting the causal relationship between imprecisely measured traits using GWAS summary data.* PLoS Genetics, 2017. **13**(11): p. e1007081.

25. Zhu, Z., et al., *Causal associations between risk factors and common diseases inferred from GWAS summary data.* Nature Communications, 2018. **9**(1): p. 1-12.

SUPPLEMENTARY APPENDIX

Eating Disorders Working Group of the Psychiatric Genomics Consortium

Roger AH Adan, PhD

Lars Alfredsson, PhD

Tetsuya Ando, MD, PhD

Ole A Andreassen, MD, PhD

Harald Aschauer, MD

Jessica H Baker, PhD

Vladimir Bencko, MD, PhD

Andrew W Bergen, PhD

Wade H Berrettini, MD, PhD

Andreas Birgegård, PhD

Joseph M Boden, PhD

Ilka Boehm, PhD

Claudette Boni, PhD

Vesna Boraska Perica, PhD

Harry Brandt, MD

Gerome Breen, PhD

Julien Bryois, PhD

Katharina Buehren, MD, PhD

Cynthia M Bulik, PhD

Roland Burghardt, MD

Laura Carlberg, MD, PhD

Matteo Cassina, MD

Sven Cichon, PhD

Maurizio Clementi, MD

Jonathan RI Coleman, PhD

Roger D Cone, PhD

Philippe Courtet, MD, PhD

Steven Crawford, MD

Scott Crow, MD

James J Crowley, PhD

Unna N Danner, PhD

Oliver SP Davis, MSc, PhD

Martina de Zwaan, MD

George Dedoussis, PhD

Daniela Degortes, PhD

Janiece E DeSocio, PhD, RN, PMHNP-BC

Danielle M Dick, PhD

Dimitris Dikeos, MD

Christian Dina, PhD

Monika Dmitrzak-Weglarz, PhD

Elisa Docampo Martinez, MD, PhD

Laramie E Duncan, PhD

Philibert Duriez, MD

Karin Egberts, MD

Stefan Ehrlich, MD, PhD

Geòrgia Escaramís, PhD

Tõnu Esko, PhD

Thomas Espeseth, PhD

Xavier Estivill, MD, PhD

Anne Farmer, MD

Angela Favaro, MD, PhD

Fernando Fernández-Aranda, PhD

Manfred M Fichter, MD, Dipl-Psych

Krista Fischer, PhD

James AB Floyd, PhD

Manuel Föcker, MD

Lenka Foretova, MD, PhD

Andreas J Forstner, MD

Monica Forzan, PhD

Christopher S Franklin, PhD

Steven Gallinger, MD

Giovanni Gambaro, MD, PhD

Héléna A Gaspar, PhD

Ina Giegling, PhD

Paola Giusti-Rodríquez, PhD

Fragiskos Gonidakis, MD

Scott Gordon, PhD

Philip Gorwood, MD, PhD

Monica Gratacos Mayora, MD, PhD

Jakob Grove, PhD

Sébastien Guillaume, MD, PhD

Yiran Guo, PhD

Hakon Hakonarson, MD, PhD

Katherine A Halmi, MD

Ken B Hanscombe, PhD

Konstantinos Hatzikotoulas, MD, PhD

Joanna Hauser, MD, PhD

Johannes Hebebrand, MD, PhD

Sietske G Helder, PhD

Stefan Herms, MSc

Beate Herpertz-Dahlmann, MD

Wolfgang Herzog, MD

Anke Hinney, PhD

L. John Horwood, MSc

Christopher Hübel, MD, PhD

Laura M Huckins, PhD

James I Hudson, MD, ScD

Hartmut Imgart, MD

Hidetoshi Inoko, PhD

Vladimir Janout, PhD

Susana Jiménez-Murcia, PhD

Craig Johnson, PhD

Jennifer Jordan, PhD

Antonio Julià, PhD

Gursharan Kalsi, PhD

Deborah Kaminská, PhD

Allan S Kaplan, MSc, MD, FRCP(C)

Jaakko Kaprio, MD, PhD

Leila Karhunen, PhD

Andreas Karwautz, MD

Martien JH Kas, PhD

Walter Kaye, MD

James L Kennedy, MD, FRCP(C)

Martin Kennedy, PhD

Anna Keski-Rahkonen, MD, PhD, MPH

Kirsty Kiezebrink, PhD, FHEA, RNutr

Youl-Ri Kim, MD, PhD

Katherine M Kirk, PhD

Lars Klareskog, MD

Kelly L Klump, PhD

Gun Peggy S Knudsen, PhD

Maria C La Via, MD

Mikael Landén, MD, PhD

Janne T Larsen, PhD

Stephanie Le Hellard, PhD

Virpi M Leppä, PhD

Robert D Levitan, MD

Dong Li, PhD

Paul Lichtenstein, PhD

Lisa Lilenfeld, PhD

Bochao Danae Lin, PhD

Jolanta Lissowska, PhD

Astri Lundervold, PhD

Jurjen Luykx, MD PhD

Pierre J Magistretti, MD, PhD

Mario Maj, MD, PhD

Katrin Mannik, PhD

Sara Marsal, MD, PhD

Christian R Marshall, PhD

Nicholas G Martin, PhD

Manuel Mattheisen, MD

Morten Mattingsdal, PhD

Sara McDevitt, MB, MD, MRCPsych, MMedED

Peter McGuffin, MD, CBE, FMedSci

Sarah E Medland, PhD

Andres Metspalu, PhD, MD

Ingrid Meulenbelt, PhD

Nadia Micali, MD, PhD

James Mitchell, MD

Karen Mitchell, PhD

Palmiero Monteleone, MD

Alessio Maria Monteleone, MD

Grant W Montgomery, PhD

Preben Bo Mortensen, MD, DrMedSc

Melissa A Munn-Chernoff, PhD

Benedetta Nacmias, PhD

Marie Navratilova, MUDr., PhD

Ioanna Ntalla, PhD

Catherine M Olsen, PhD, MPH

Roel A Ophoff, PhD

Julie K O'Toole, MD

Leonid Padyukov, MD, PhD

Aarno Palotie, MD, PhD

Jacques Pantel, PhD

Hana Papezova, MD, PhD

Richard Parker, BA(Hons)

John F Pearson, PhD

Nancy L Pedersen, PhD

Triinu Peters, PhD

Liselotte Petersen, PhD

Dalila Pinto, PhD

Kirstin L Purves, PhD

Anu Raevuori, MD, PhD

Nicolas Ramoz, PhD

Ted Reichborn-Kjennerud, MD, PhD

Valdo Ricca, MD

Samuli Ripatti, PhD

Stephan Ripke, MD, PhD

Franziska Ritschel, MSc

Marion Roberts, PhD

Alessandro Rotondo, MD

Dan Rujescu, MD

Filip Rybakowski, MD, PhD

Paolo Santonastaso, MD

André Scherag, PhD

Stephen W Scherer, PhD, FRSC

Ulrike Schmidt, MD, PhD

Nicholas J Schork, PhD

Alexandra Schosser, PhD

Jochen Seitz, MD

Lenka Slachtova, PhD

P. Eline Slagboom, PhD

Margarita CT Slof-Op 't Landt, PhD

Agnieszka Slopien, MD, PhD

Nicole Soranzo, PhD

Sandro Sorbi, MD

Lorraine Southam, BSc

Vidar W Steen, MD, PhD

Michael Strober, PhD

Patrick F Sullivan, MD, FRANZCP

Beata Świątkowska, PhD

Jin P Szatkiewicz, PhD

Elena Tenconi, MD

Laura M Thornton, PhD

Alfonso Tortorella, MD

Federica Tozzi, MD

Janet Treasure, MD, PhD, FRCP, FRCPsych

Artemis Tsitsika, MD, PhD

Marta Tyszkiewicz-Nwafor, MD, PhD

Konstantinos Tziouvas, MD, MSc

Annemarie A van Elburg, MD, PhD

Eric F van Furth, PhD

Tracey D Wade, PhD

Gudrun Wagner, Dr, MSc, DPO

Esther Walton, Dr. rer. nat., PhD

Hunna J Watson, PhD, MPsychClin, MBiostat

Thomas Werge, PhD

David C Whiteman, MBBS(Hons), PhD, FAFPHM

H-Erich Wichmann, PhD

Elisabeth Widen, MD, PhD

D. Blake Woodside, MD

Shuyang Yao, PhD

Zeynep Yilmaz, PhD

Eleftheria Zeggini, PhD

Stephanie Zerwas, PhD

Yiran Zheng, MSc

Stephan Zipfel, MD
